# Overexpression of ERBB4 JM-a CYT-1 and CYT-2 isoforms in transgenic mice reveals isoform-specific roles in mammary gland development and carcinogenesis

**DOI:** 10.1186/s13058-014-0501-z

**Published:** 2014-12-17

**Authors:** Vikram B Wali, Maureen Gilmore-Hebert, Ramanaiah Mamillapalli, Jonathan W Haskins, Kari J Kurppa, Klaus Elenius, Carmen J Booth, David F Stern

**Affiliations:** 1 Department of Pathology, Yale School of Medicine, P.O.Box 208023, New Haven, 06520 CT USA; 2 Department of Breast Medical Oncology, Yale Cancer Center, Room#786, 300 George Street, New Haven, CT-06511 USA; 3 Department of Medicinal Biochemistry and genetics and Medicity Research Laboratories, University of Turku, Kiinamyllynkatu 10, Turku, 20520 Finland; 4 Section of Comparative Medicine, Yale School of Medicine, P.O. Box 208016, New Haven, CT 06520 USA

## Abstract

**Introduction:**

Human Epidermal Growth Factor Receptor (ERBB4/HER4) belongs to the Epidermal Growth Factor receptor/ERBB family of receptor tyrosine kinases. While ERBB1, ERBB2 and ERBB3 are often overexpressed or activated in breast cancer, and are oncogenic, the role of ERBB4 in breast cancer is uncertain. Some studies suggest a tumor suppressor role of ERBB4, while other reports suggest an oncogenic potential. Alternative splicing of ERBB4 yields four major protein products, these spliced isoforms differ in the extracellular juxtamembrane domain (JM-a versus JM-b) and cytoplasmic domain (CYT-1 versus CYT-2). Two of these isoforms, JM-a CYT-1 and JM-a CYT-2, are expressed in the mammary gland. Failure to account for isoform-specific functions in previous studies may account for conflicting reports on the role of ERBB4 in breast cancer.

**Methods:**

We have produced mouse mammary tumour virus (MMTV) -ERBB4 transgenic mice to evaluate potential developmental and carcinogenic changes associated with full length (FL) JM-a ERBB4 CYT-1 versus ERBB4 CYT-2. Mammary tissue was isolated from transgenic mice and sibling controls at various developmental stages for whole mount analysis, RNA extraction, and immunohistochemistry. To maintain maximal ERBB4 expression, transgenic mice were bred continuously for a year after which mammary glands were isolated and analyzed.

**Results:**

Overexpressing FL CYT-1 isoform resulted in suppression of mammary ductal morphogenesis which was accompanied by decreased number of mammary terminal end buds (TEBs) and Ki-67 positive cells within TEBs, while FL CYT-2 isoform had no effect on ductal growth in pubescent mice. The suppressive ductal phenotype in CYT-1 mice disappeared after mid-pregnancy, and subsequent developmental stages showed no abnormality in mammary gland morphology or function in CYT-1 or CYT-2 transgenic mice. However, sustained expression of FL CYT-1 isoform resulted in formation of neoplastic mammary lesions, suggesting a potential oncogenic function for this isoform.

**Conclusions:**

Together, we present isoform-specific roles of ERBB4 during puberty and early pregnancy, and reveal a novel oncogenic property of CYT-1 ERBB4. The results may be exploited to develop better therapeutic strategies in breast cancer.

**Electronic supplementary material:**

The online version of this article (doi:10.1186/s13058-014-0501-z) contains supplementary material, which is available to authorized users.

## Introduction

ERBB4/human epidermal growth factor receptor 4, the fourth member of the epidermal growth factor receptor (EGFR) family, is predominantly expressed in the heart, brain, kidney, salivary glands, and mammary glands [1]. In contrast to EGFR and ErbB2, which are expressed and activated in mouse mammary glands at puberty, ErbB4 is mainly active during pregnancy and lactation [2],[3]. The critical role of ErbB4 expression in pregnant and lactating mammary gland development was established using loss-of-function strategies. ErbB4 signaling is necessary for terminal mammary differentiation and for signal transducer and activator of transcription 5 (Stat5) activation late in pregnancy and during lactation [4], and homozygous loss of function leads to defects in pregnancy and lactation [5]. Additionally, ErbB4 and its ligand Nrg3 have been implicated in mammary bud specification in mouse embryos [6]. We have previously shown that Neuregulin 1 (NRG1), a ligand for ErbB4 and ErbB3, induces proliferation and differentiation of mammary epithelium in prepubescent mice, indicating the presence of functional ErbB4 or/and ErbB3 at early developmental stages [7]. However, the exact roles of ERBB4 in mammary gland development in nulliparous mice are not fully understood.

*ErbB4* is unique in the EGFR family in that it yields multiple alternatively spliced mRNA isoforms, and the protein products undergo regulated extracellular and intramembrane proteolysis. The spliced isoforms differ in the extracellular juxtamembrane domain (JM-a vs. JM-b isoforms) and the cytoplasmic domain (CYT-1 vs. CYT-2), and their relative expression varies with tissue type. The JM-a and JM-b isoforms use alternate exons that encode sequences just outside the transmembrane domain, with the JM-a sequences including a tumor necrosis factor alpha-converting enzyme metalloproteinase cleavage site rendering JM-a but not JM-b isoforms, susceptible to cleavage. Tumor necrosis factor alpha-converting enzyme cleavage of the JM-a isoform releases the extracellular domain, leaving membrane-associated 80 kDa (m80) truncated ERBB4. This undergoes a secondary presenilin/γ-secretase-dependent intramembrane cleavage, releasing a constitutively kinase-active soluble intracellular domain (ICD), s80, which translocates to the nucleus and regulates transcription [8],[9]. The JM-c isoform lacking sequences from both exons and the JM-d isoform with both exon-encoded sequences have also been reported. Cytoplasmic isoforms CYT-1 and CYT-2 differ in that 16 amino acids present in CYT-1 are absent in CYT-2 as a result of exon skipping in the latter. This 16 amino acid peptide includes consensus binding sites for WW domains and for the SH2 domain of the p85 subunit of phosphatidyl-inositol (3′)-kinase, and hence CYT-1 can activate the phosphatidyl-inositol (3′)-kinase–Akt pathway [10]. The WW domain-containing ubiquitin E3 ligase Aip4/Itch binds to the PPXY1056 Itch binding site present only in CYT-1, resulting in higher ligand-induced ubiquitination of CYT-1 than that of CYT-2 [11]. Normal mammary glands and breast cancers express cleavable JM-a isoforms, CYT-1 and CYT-2 but not JM-b.

*ERBB4* is one of the top 127 significantly mutated genes across 12 cancers [12]. Potential oncogenic mutations in the protein tyrosine kinase domain and elsewhere in ERBB4 have been reported for melanoma, gastric carcinoma, colorectal carcinoma, nonsmall-cell lung carcinoma, and breast carcinoma, but most have not been functionally validated. ERBB4 mutations are infrequent in breast cancer, with a prevalence of approximately 1% [12], and *ERBB4* gene amplification is rare [13],[14]. *ERBB4*, predominantly the CYT-1 isoform, is overexpressed in medulloblastoma [15]. Correlative biomarker studies have implied either pro-tumorigenic or anti-tumorigenic activity of ERBB4 in breast cancer [16],[17]. As very different biological activities are induced in tissue culture by CYT-1 and CYT-2 isoforms, and as only JM-a ERBB4 can be cleaved to yield the nuclear form, it is possible that the impact of ERBB4 varies considerably depending on isoform or cleavage. *In vitro*, the ICD of ERBB4 suppresses proliferation and induces differentiation, but, interestingly, ribozyme-mediated ERBB4 downregulation and use of antibody against cleavable ERBB4 has also been shown to suppress tumor cell proliferation [18]-[20]. In one breast cancer study, high expression of ERBB4 was associated with a favorable outcome in estrogen receptor-positive cases; in the same study, nuclear ERBB4 immunoreactivity was associated with poor survival as compared with women whose cancer had membranous ERBB4 staining [21]. Nuclear ERBB4 ICD is inversely correlated with tumor grade and tumor mitosis, while cytosolic ERBB4 ICD has significant positive prognostic value in lymph node-negative breast cancer patients [22].

In tissue culture, JM-a CYT-1 and CYT-2 ERBB4 isoforms exhibit a range of cellular functions depending on the cell type and the receptor model system studied; that is, whether full length (FL) or artificially truncated (ICD) receptors are investigated. The CYT-1 isoform has antiproliferative activity in SUM102 mammary cancer cells, 32D bone marrow cells, and HC11 and MCF10A mammary epithelial cells, while CYT-1 ERBB4 promotes tumorigenesis in ovarian OVCAR-3 and SKOV-3 cancer cell lines [23]-[27]. In mice, expression of sequences encoding the s80 CYT-1 ICD decreased mammary ductal growth with no effect on lobuloalveolar growth whereas CYT-2 caused mammary epithelial hyperplasia [23]. While this study was important in revealing major differences between CYT-1 and CYT-2 isoforms in the mouse mammary gland, these observations were made with a constitutively active truncated *ErbB4* that in tissue culture has greater signaling power and possibly different signaling targets from the FL molecule [25]. *In vivo*, the signaling activity of ErbB4 isoforms will be a composite of intact ErbB4 signaling, through recruitment of signaling proteins to noncleaved ERBB4 embedded in cellular membranes, and through the very different signaling qualities associated with constitutively active soluble ERBB4, much of which homes to the nucleus. Moreover, the biological activities will be modulated by endogenous activation of metalloproteinases and γ-secretase activities. To address the *in vivo* signaling properties of intact ErbB4 isoforms expressed in transgenic animals, we produced a gain-of-function transgenic mouse model that overexpresses FL CYT-1 and CYT-2 JM-a *ERBB4* human transgenes driven by the mouse mammary tumor virus (MMTV) promoter/enhancer sequences, in order to evaluate the potential developmental and carcinogenic roles of *ERBB4* CYT-1 and CYT-2. This model system was used to address uncertainties about the role of each FL ERBB4 isoform in nulliparous and parous mammary gland development and the long-term effect of each isoform on tumorigenesis.

## Materials and methods

### Transgenic mice

**Figure 1:**
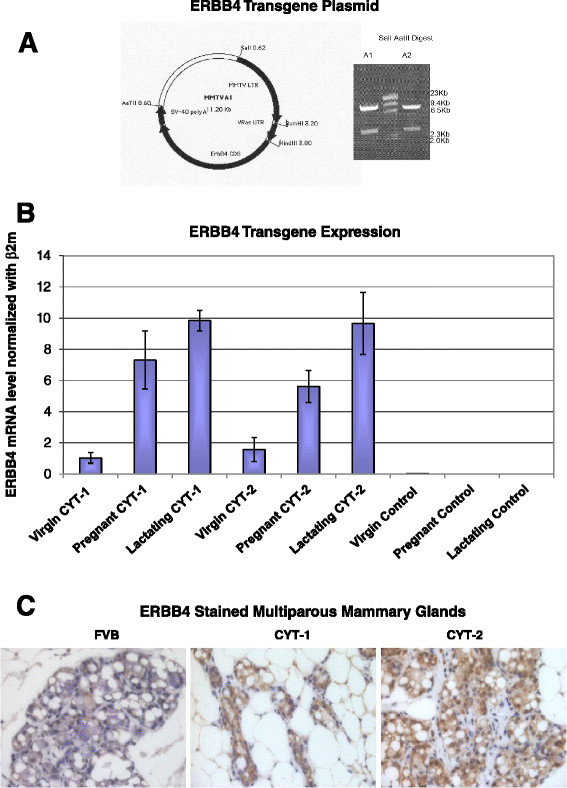
**ERBB4 transgene expression. (A)** Plasmid map for MMTV-ERBB4 carrying SalI and AatII sites. SalI-AatII double digestion of the plasmid separated the ERBB4 CYT-1(A1) and CYT-2 (A2) inserts, which were gel purified for micro-injections to generate CYT-1 and CYT-2 ERBB4 transgenic mice. **(B)** ERBB4 mRNA expression in the mammary glands of CYT-1 and CYT-2 mice at various developmental stages. RNA was extracted from mammary glands from three mice at each stage and pooled. Relative ERBB4 expression levels are represented by vertical bars as measured by real-time quantitative reverse transcription PCR using Taqman primers, with error bars indicating the standard deviation for samples run in triplicate. **(C)** ERBB4 immunohistochemical staining (detecting both mouse endogenous and human transgenic ERBB4) in nonpregnant multiparous female transgenic and control FVB mice. β2m, mouse-specific beta-2 microglobulin; MMTV, mouse mammary tumor virus.

Plasmids encoding human ERBB4 isoforms JM-a CYT-1 ERBB4 and JM-a CYT-2 ERBB4, under control of the MMTV long terminal repeat, the v*RAS* 5′-untranslated region, human ERBB4 cDNAs, and the SV40 polyadenylation site were produced by ligating the insert fragments ERBB4 JM-a CYT-1 and ERBB4 JM-a CYT-2 from pcDNA3.1 into digested pMMTV-erbB4∆IC as vector. Briefly, pMMTV-erbB4∆IC was digested with EcoRI, blunted using T4 polymerase and digested again with BstEII, and the resulting 8,300 base pair vector fragment was isolated from an agarose gel and spin-column purified. pcDNA3.1.ERBB4JM-aCYT-1 and pcDNA3.1.ERBB4JM-aCYT-2 were digested with BstEII and PmeI, and the resulting JM-a CYT-1 and JM-a CYT-2 fragments were isolated from an agarose gel, spin-column purified, and ligated into digested pMMTV. Inserts from the final constructs were confirmed by DNA sequencing. The insert separated by SalI-AatII digestion was gel purified (Figure 1A) and micro-injected into fertilized ova from FVB females, which were implanted into pseudopregnant CD1 female mice by the Yale Animal Genomics Services core (New Haven, CT, USA). DNA from tail biopsies of offspring was genotyped for ERBB4 using PCR primers 5′-CTGGTCATTGTGGGTCTGAC, corresponding to nucleotides 2,088 to 2,107 of exon 17, and 5′-CTCCTTCCAAGAGTCTGGCT, the reverse complement corresponding to nucleotides 2,690 to 2,709 of exon 21 of *ERBB4*. Transgene-positive mice were backcrossed with FVB mice and their progeny were tested for transmission of the transgene. Multiple lines were found to express the transgene. In this study, lines L2 for CYT-1, and V12 for CYT-2 were used for detailed analysis. The transgenes were inherited according to Mendelian expectations for heterozygotes. All animal work was approved by Yale University Institutional Animal Care and Use Committee and followed internationally recognized guidelines.

### RNA extraction and ERBB4 transgene expression

Mammary tissue from transgenic mice and sibling controls was isolated at various developmental stages, and stored immediately in RNAlater (Qiagen; Valencia, CA, USA). The tissue was homogenized and total RNA was isolated with the RNeasy Plus Mini Kit (Qiagen) and reverse-transcribed with the iScript cDNA Synthesis Kit (BioRad; Hercules, CA, USA), using 1 μg RNA per reaction. Universal TaqMan Master Mix (Applied Biosystems; Grand Island, NY, USA) was used for real-time quantitative reverse transcription PCR analysis of a 1:10 dilution of the resulting cDNA. *ERBB4* transgene mRNA expression was quantified by real-time quantitative reverse transcription PCR using Taqman primers (Applied Biosystems) for *ERBB4* (Hs00171783_m1) and mouse-specific beta-2 microglobulin (Mm03003532_u1) according to the manufacturer’s protocols. Relative mRNA expression was determined with the ΔCt method, with mouse-specific beta-2 microglobulin as the reference gene. The ERBB4 antibody (sc-283) used for immunohistochemistry (IHC) binds to the intracellular region near the carboxyl terminus of ERBB4, and reacts with both mouse and human ERBB4.

### Whole mount staining and terminal end bud count

Left-side #4 mammary glands were isolated from female mice for whole mount staining with Carmine Alum. Briefly, mammary glands were air dried for 10 to 15 minutes on a clean glass slide, and fixed in Carnoy’s fixative (75% ethanol + 25% acetic acid). Slides were then washed in 70% ethanol, rinsed in water, and stained overnight with Carmine Alum, followed by sequential dehydration steps in 70%, 95% and 100% ethanol. Afterward, glands were defatted in acetone, cleared in xylene and mounted with Permount and coverslipped. Terminal end buds (TEBs) in the Carmine Alum-stained mammary whole mount slides were counted manually under magnification.

### Quantification of mammary ductal morphogenesis

Entire glands were photographed under a dissection microscope with a SPOT 11.2 Color Mosaic camera (Diagnostic Instruments Inc; Sterling Heights, MI, USA) at 10× magnification using SPOT advanced software 4.0.9 (Diagnostic Instruments Inc; Sterling Heights, MI, USA) (Figures S1 to S8 in Additional file 1). Images were analyzed and quantified with ImageJ software (Wayne Rasband, National Institutes of Health, Bethesda, Maryland, USA). Ductal growth was calculated as the percentage of the distance from the lymph node to the farthest point of the longest duct relative to the distance to the farthest limit of the mammary fat pad.

For measuring ductal branching in virgin mice, branch points were counted manually in three random unit areas using ImageJ software. At each developmental stage, between three and 12 mice were quantified in both sibling control and transgenic groups. Statistical analysis was performed using one-way analysis of variance using Newman–Keuls post-analysis of variance multivariate analysis. Post-coitus whole mount mammary glands were blinded and scored over a range of 1 to 10 for highest to lowest branching density after microscopic inspection.

High-resolution individual and composite digital light microscopic representative images of developing mammary glands at 5, 8, and 14 weeks from control, CYT-1 and CYT-2 mice were recorded using an Axio Imager.A.1 microscope with an AxioCam MRc5 camera and AxioVision 4.7 imaging software (Zeiss; Thornwood, NJ, USA). Images were optimized in Adobe Photoshop CS5 12.0.4 (Adobe Systems Inc.; San Jose, CA, USA).

### Evaluation of mammary tumor lesions

Mammary lesions were identified by gross examination of mammary glands and stained by carmine alum in the whole mount slides. These mammary glands were paraffin embedded and thin sections were stained with hematoxylin and eosin (H&E). Microscopic pathological examination of these H&E slides was performed and the lesions were categorized according to their histological characteristics (Additional file 2).

### Hematoxylin and eosin, Masson trichrome stain and immunohistochemistry

A portion of the right #4 inguinal mammary gland was spread onto a glass microscope slide and fixed in freshly prepared 4% paraformaldehyde in phosphate-buffered saline overnight. The fixed tissue was embedded in paraffin, and 5 μm sections were dried onto gelatin-coated slides. Samples were then deparaffinized and rehydrated in distilled water. Endogenous peroxidase was quenched with hydrogen peroxide. Masson trichrome and H&E staining were performed according to standard methods.

Primary antibodies ErbB4 and Stat5a (Santa Cruz Biotechnology; Dallas, Texas, USA), p63, CK5/6 and proliferating cell nuclear antigen (PCNA; Dako, Carpinteria, CA, USA), Vimentin, E-cadherin and smooth muscle actin (Thermo Scientific, Waltham, MA, USA), and Ki-67 (Biocare Medical, Concord, CA, USA) were used for immunohistochemistry on formalin-fixed paraffin-embedded sections. Immunoreactivity was detected with the peroxidase-based Envision + system (Dako). Diaminobenzidine was used to detect the antibody complex (Dako). The slides were subsequently counterstained with hematoxylin and then dehydrated and permanently coverslipped in resin mounting media. For PCNA quantification in TEBs, the total number of TEBs (positive and negative for PCNA) per section (*n* = 4 for each control and transgenic mice) was counted under the microscope; within each TEB positive for PCNA, the percentage of PCNA-positive cells was calculated by counting PCNA-positive versus all luminal TEB cells.

## Results

### ERBB4 expression during development

To compare biological activities of ERBB4 CYT-1 and CYT-2, we produced transgenic mice in which human ERBB4 cDNAs are expressed under control of the MMTV promoter/enhancer (Figure 1A). Since the genomic integration site affects the expression levels of transgenes driven by the MMTV promoter, ERBB4 expression in mammary glands was assessed in multiple mice lines by real-time quantitative reverse transcription PCR, and lines with higher expression (L1 in CYT-1 group and V12 in CYT-2) in virgin mice were used for further analysis. ERBB4 expression was detected by real-time quantitative reverse transcription PCR in virgin, post-coitus and post-partum female transgenic mice (Figure 1B). In FVB mice, MMTV-driven transgenes are expressed mainly in the mammary epithelium, and usually at low levels in young animals, with higher expression in pregnancy and lactation. As expected, expression of ERBB4 CYT-1 and CYT-2 transgenes was significantly higher in pregnant (12 days post coitus) and lactating mice and 1-day post-partum females compared with pubescent 5-week-old mice. Similar expression trends across developmental stages were seen in both CYT-1 and CYT-2 transgenic mice; direct comparison between mice harboring the two transgenes at each stage revealed comparable expression levels. ERBB4 was not detectable by IHC in virgin mice, but ErbB4 was readily detected by IHC in multiparous nonpregnant female mice (Figure 1C). Staining intensity and number of ERBB4 positively stained cells was clearly higher in transgenic mice mammary glands as compared with background FVB mice (Figure 1C).

### Transgenic ERBB4 expression effect on mammary gland development

**Figure 2:**
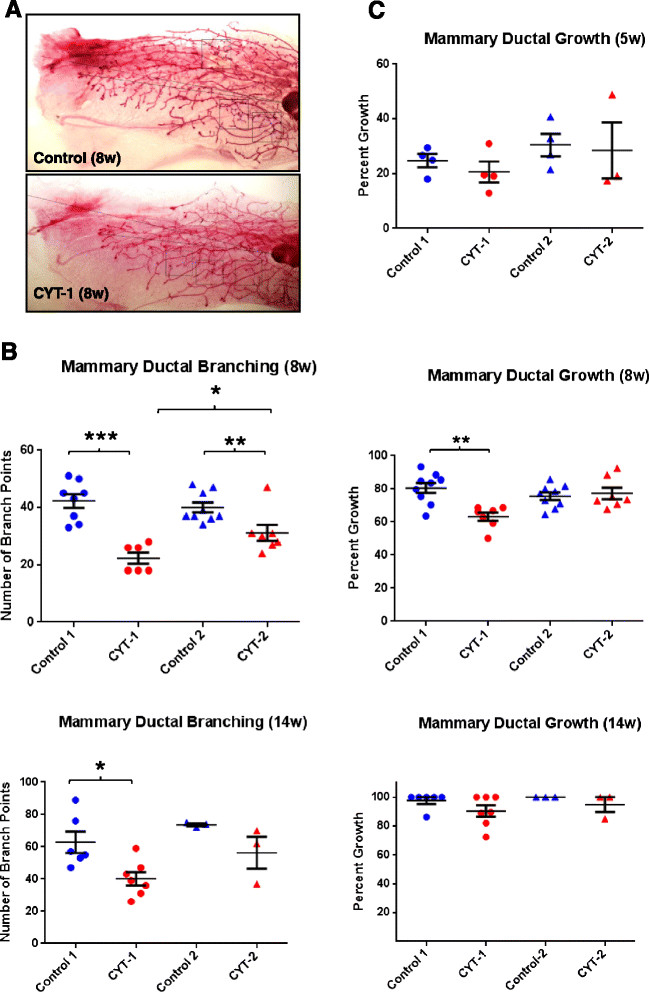
**Quantitative analysis of mammary ductal elongation and branching.** Mammary gland #4 was isolated from 5-week-old, 8-week-old and 14-week-old (5w, 8w and 14w) CYT-1 (red circle) and CYT-2 (red triangle) ERBB4 transgenic mice and their respective transgene negative sibling controls (blue circles and triangles), and was processed for whole mount analysis using Carmine Alum stain. **(A)** Stained whole mounts were photographed under a dissection microscope with a SPOT 11.2 Color Mosaic camera at 10× magnification using SPOT advanced software 4.0.9 (Diagnostic Instruments Inc; Sterling Heights, MI, USA). Ductal growth and branching were measured using ImageJ software (National Institute of Health, Bethesda, Maryland, USA). **(B)** For ductal branching, branch points were counted manually in three random unit areas using ImageJ software and averaged for each mouse. Data points indicate branch points ± standard error of the mean (SEM) in each group. **(C)** Ductal elongation/growth was calculated as the percentage of the distance from the lymph node to the farthest point of the longest duct relative to the distance to the farthest limit of the mammary fat pad. Data points indicate percent growth ± SEM in each group. ***P <0.001, **P <0.01, *P <0.05 by one-way analysis of variance (ANOVA) using Newman–Keuls post-ANOVA multivariate analysis.

**Figure 3:**
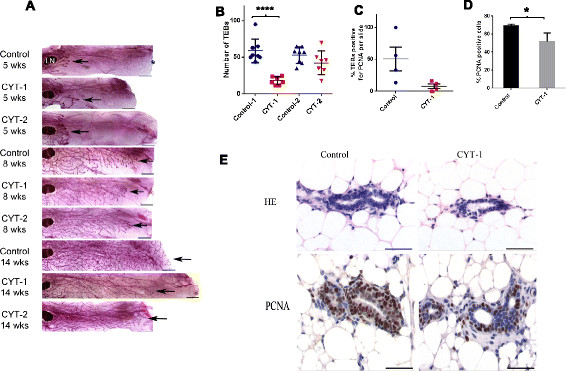
**Quantification of terminal end buds and proliferating cell nuclear antigen staining. (A)** High-resolution composite digital light microscopic representative images of developing mammary glands at 5, 8, and 14 weeks from control, CYT-1 and CYT-2 mice were recorded using an Axio Imager.A.1 microscope with an AxioCam MRc5 camera and AxioVision 4.7 imaging software (Zeiss, Thornwood, NJ, USA). LN, lymph node. *End of the gland area, arrowheads point to the end of the terminal end buds (TEBs). Scale bars = 1,000 μm. **(B)** The number of TEBs in 8-week-old mammary gland whole mount slides was counted manually under magnification in transgenic CYT-1 and CYT-2 mice and their respective sibling controls. ****P <0.0001 by one-way analysis of variance (ANOVA) using Tukey’s post-ANOVA multivariate analysis. **(C)** Proliferating cell nuclear antigen (PCNA)-positive TEBs (TEBs with at least one luminal cell displaying positive PCNA nuclear staining) and total number of TEBs in thin mammary tissue sections of 8-week-old CYT-1 mice and sibling controls were counted, and expressed as percent positive for PCNA. **(D)** Percent PCNA-positive cells were calculated for each PCNA-positive TEB within a section of 8-week mammary gland in four mice in each control and CYT-1 and were averaged. *P <0.05 by unpaired *t* test. **(E)** Representative hematoxylin and eosin (HE) and PCNA stained images of TEBs in 8-week mammary glands. Scale bars = 50 μm.

EGFR, ErbB2, and ErbB3 are required for normal mouse mammary development at puberty, but ErbB4 has mainly been implicated in pregnancy and in lactation [4],[28]-[32]. At 5 weeks of age, no differences were evident between mammary ducts of control and ERBB4 transgenic female mice, which traverse approximately 25 to 30% of the fat pad by this time. However, at 8 weeks, mammary ductal growth and branching of ERBB4 CYT-1 mice was significantly lower than that of sibling controls as well as that of ERBB4 CYT-2 mice, with approximately 60% average ductal penetration of the fat pad in CYT-1 mice as compared with ~80% growth in other groups (Figures 2A,B,C and 3A). Moreover, ductal branching density of ERBB4 CYT-1 mice (approximately 20 branches per unit area) was approximately one-half that of sibling controls (approximately 40 branches per unit area) (Figures 2B and 3A). Although mammary ductal growth of CYT-2 mice was not significantly different from sibling controls, CYT-2 mammary ductal branching was significantly decreased at 8 weeks (Figures 2B and 3A). In adult females 14 weeks of age, mammary ducts in all of the groups nearly filled the mammary fat pad (100% ductal growth). Ductal branching in 14-week-old CYT-2 mice was at or near the levels of sibling controls, but branching in CYT-1 mice remained significantly lower than the controls (Figures 2B and 3A). TEBs were evident in whole mounts of 5-week and 8-week mammary glands, but had regressed by 14 weeks (Figure 3A). There was no significant difference between 5-week control and transgenic groups, but a significantly lower number of TEBs (average ~18) was found in CYT-1 whole mount mammary gland slides as compared with control (average ~61) (Figure 3B). Immunostaining of PCNA in thin sections revealed a large variation in the percentage of TEBs with PCNA-positive nuclei as compared with the CYT-1 group, but these differences were not statistically significant (Figure 3C). Within each PCNA-positive TEB (with at least one cell with positive PCNA nuclear staining), the percentage of PCNA-positive cells was lower in the CYT-1 group (Figure 3D,E). Decreased mammary ductal growth and branching in 8-week-old CYT-1 mice was thus associated with decreased proliferating cells within the TEBs. H&E staining of TEBs of 8-week-old CYT-1 mice also suggested a possible defect in the architecture of CYT-1 TEBs, as suggested by an apparent decrease in body cell number and larger luminal spaces compared with controls (Figure 3E).

**Figure 4:**
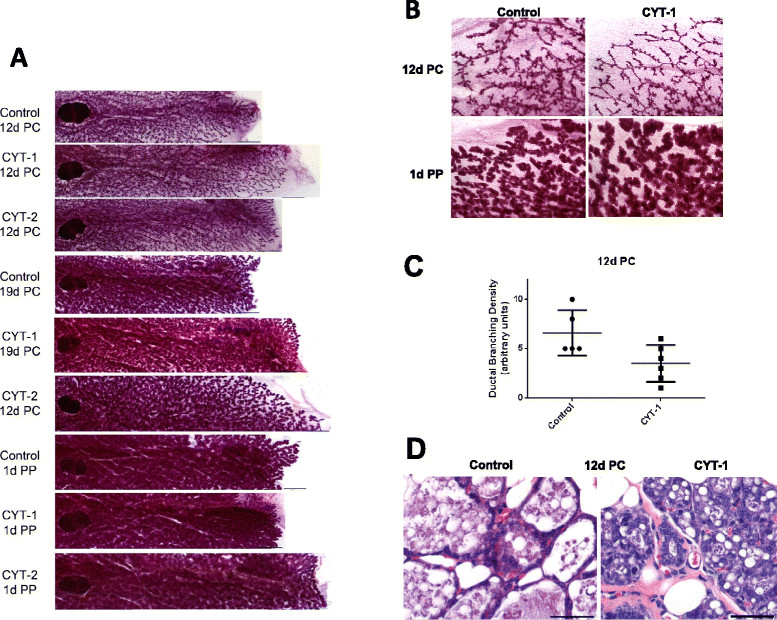
**Mammary ductal branching density in post-coitus mice. (A)** High-resolution composite digital light microscopic representative images of 12-days post-coitus (12d PC) and 1 day post-partum (1d PP) control, CYT-1, and CYT-2 mice were recorded using an Axio Imager.A.1 microscope with an AxioCam MRc5 camera and AxioVision 4.7 imaging software (Zeiss, Thornwood, NJ, USA), or **(B)** a SPOT 11.2 Color Mosaic camera (Diagnostic Instruments Inc; Sterling Heights, MI, USA) for a magnified image. **(C)** 12d PC whole mount mammary glands were blinded and scored over a range of 1 to 10 for highest to lowest branching density after microscopic inspection. Data points indicate percent growth ± standard deviation in each group *P <0.05 by unpaired t test. **(D)** Representative hematoxylin and eosin (HE) image of control and CYT-1 PC-12 mammary gland thin section. Scale bars = 50 μm.

In pregnant mice 12 days post coitus, mammary gland ductal branching continued to be less dense in CYT-1 transgenic mice than for CYT-2 or control animals (Figure 4A,B,C). This was confirmed with H&E staining, which additionally indicated underdeveloped milk glands in CYT-1 mice, as controls had larger number of large acini and more flattened epithelial cells (Figure 4D). However, at 19 days post coitus, 1 day post partum, 16 days post partum, and 16 days post weaning, mammary whole mounts and H&E histology of thin sections revealed no major differences in mammary gland morphology among control and transgenic groups (Figures S1 to S8 in Additional file 1). Interestingly, multiparous CYT-1 mammary glands appear morphologically distinct from the FVB control and CYT-2 mammary glands with, fewer lobuloalveolar structures. This is consistent with a growth inhibitory role of CYT-1 ERBB4 during early developmental stages.

### Tumorigenesis in ERBB4 transgenic mice

**Figure 5:**
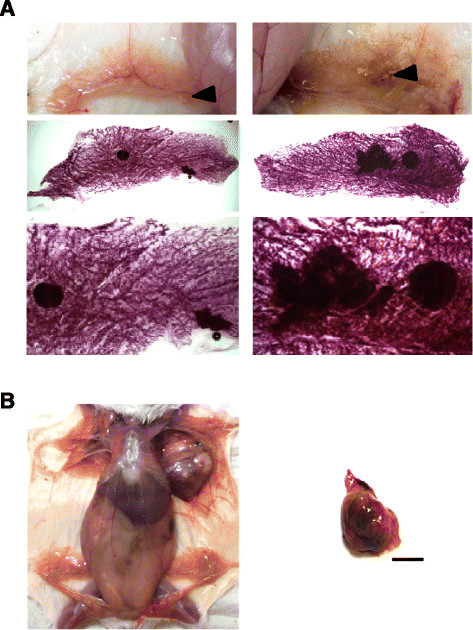
**Mammary tumorigenesis in mouse mammary tumor virus CYT-1 ERBB4 transgenic mice.** Mammary tumor lesions observed in 52-week-old multiparous CYT-1 ERBB4 transgenic mice. **(A)** Low-magnification bright-field image (top) before whole mount analysis, and low-magnification (middle) and high-magnification (bottom) bright-field image after whole mount analysis in the same female CYT-1 mouse. **(B)** Low-magnification bright-field image before whole mount analysis of another age-matched female CYT-1 ERBB4 mouse (left), and tumor separated from the same mouse (right). Scale bar = 1 cm.

The role of ERBB4 in breast cancer is uncertain. To promote chronic ERBB4 signaling through overexpression with maximal activity of the MMTV promoter, we maintained 12 CYT-1 females and 12 CYT-2 females in continuous breeding with nontransgenic control males. Six control female FVB mice were continuously bred separately. At the end of approximately 1 year (52 weeks), these multiparous female breeders were euthanized for analysis. Mammary gland whole mounts revealed single or multiple tumor lesions in 10 out of 12 CYT-1 mice (Figure 5A). Two out of 12 of the CYT-2 group showed hyperplastic regions, whereas no significant findings were observed in the control group (0/6). One mouse in the CYT-1 group also developed a 2.0 cm × 2.0 cm solid mammary tumor (Figure 5B) in addition to multiple smaller mammary lesions. The MMTV promoter has been used extensively in mammary tumor mouse models to study the phenotypes of transgenes in the FVB mouse background. These include wild-type *neu/ErbB2* transgenic mice with tumor latency of 30 to 52 weeks, and MMTV-cyclin D_1_ mice with approximately 70 weeks as mean age at onset [33],[34]. Spontaneous mammary hyperplasia and tumor incidence have been reported in multiparous female FVB mice, but with a latency period >80 weeks [35].

Lesions were first identified by visual inspection while isolating mammary glands, and they were conspicuous in carmine alum-stained whole mounts. Paraffin-embedded thin sections stained with H&E were evaluated microscopically. Pathological examination of these H&E slides characterized these identified lesions as neoplastic or hyperplastic (Additional file 2).

### Characteristics of tumor lesions

**Figure 6:**
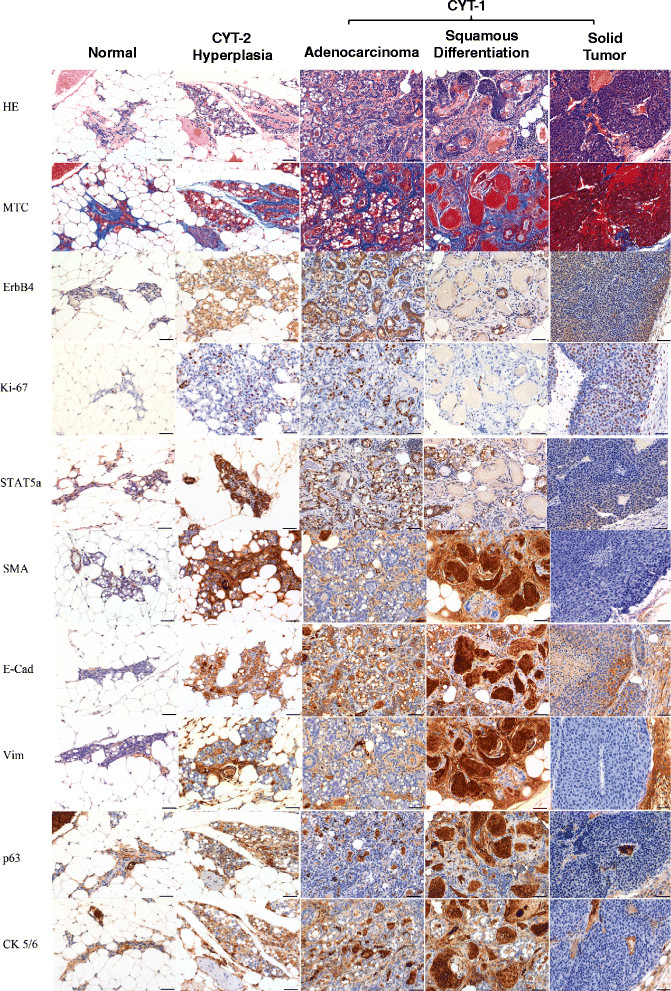
**Immunohistochemical analysis of mammary tissue in serial sections.** Immunohistochemistry images of the mammary tissue isolated from age-matched (52-week) female multiparous control FVB (normal) mice, and abnormal regions observed in CYT-2 (hyperplasia) and CYT-1 (adenocarcinoma, squamous differentiation, solid tumor) ERBB4 transgenic mice. Stained representative images of the mouse mammary tissue obtained from FVB control (*n* = 3), CYT-2 with hyperplastic region (*n* = 1) and CYT-1 (*n* = 3) carcinoma, and a single 2 cm × 2 cm CYT-1 solid tumor. Tissues were embedded in paraffin sections and stained for hematoxylin and eosin (HE), Masson trichrome (MTC; stains for collagen (blue), nuclei (black) and muscle/keratin (red)), ErbB4, cell proliferation marker Ki-67, signal transducer and activator of transcription 5a (STAT5a), smooth muscle actin (SMA; stains for smooth muscle and myoepithelium), epithelial markers E-Cadherin (E-Cad) and cytokeratin 5/6 (CK5/6), and mesenchymal markers Vimentin (Vim) and p63. Scale bars = 50 μm.

Histopathological examination of H&E-stained and immunostained slides showed that tumor lesions from ERBB4 CYT1 mice showed similar characteristics, with regions of hyperplasia or carcinoma with glandular or squamous differentiation or solid tumor (Figure 6; Additional file 2). Non-neoplastic mammary gland regions in transgenic mice were structurally similar to those of nontransgenic mice. Similar morphology for all of the tumor lesions suggests a common underlying cause for neoplastic transformation in CYT-1 mice. Interestingly, the hyperplastic regions observed in two CYT-2 mice were very similar to those seen in all CYT-1 mammary lesions, suggesting a qualitative overlap in the tumorigenic processes between CYT-1 and CYT-2 ERBB4 transgenic mice. Although hyperplastic regions in CYT-2 mice qualitatively appear to be a subset of the CYT-1 phenotype, they occurred with low incidence (only 2/12) and so are difficult to compare quantitatively with the more penetrant CYT-1 lesions (10/12). Gross examination of tissue did not reveal overt signs of metastatic lesions in the liver, lungs, kidneys, or brain. Morphologically, MMTV-CYT-1 adenocarcinomas do not resemble *MMTV-Neu* and *MMTV-PyVmT* tumors. CYT-1 also differs from these tumors in latency period (4 months, *MMTV-Neu*; 5 weeks, *MMTV-PyVmT*) and because no tumors develop in virgin CYT-1 mice in contrast to the other two tumor models. Although there is expansion of the basal compartment in CYT-1 adenocarcinoma, the single large tumor observed is more similar to these models. However, CYT-1 tumors somewhat resemble slow-growing MMTV-cyclin D_1_ tumors (with latency of approximately 500 days), which are characterized by squamous differentiation as seen in CYT-1 [33],[34].

### Breast carcinomas express ERBB4 that correlates with STAT5a and Ki-67 staining

ERBB4 protein was stained by IHC with an antibody that reacts with both mouse and human ERBB4. IHC of mammary tissues showed that ErbB4 levels were higher in transgenic mice as compared with nontransgenic mice (Figure 1C). Figure 6 shows the histopathology of mammary tissue derived from normal (nontransgenic) mice, hyperplastic (CYT-2) mice, and carcinoma with glandular and squamous cell differentiation (CYT-1 mice). These microscopic images are representative of multiple thin sections obtained from three FVB control mice, three CYT-1 mice (carcinoma), and one CYT-2 mouse with a hyperplastic region. One mouse had a solid mammary carcinoma lacking both glandular and squamous cell differentiation (CYT-1 mouse). Within the transgenic mammary tissue, ERBB4 cytoplasmic staining was observed (50 to 100%) in CYT-1 glandular cells and in squamous tumor epithelial cells with scattered positive cytoplasm (~25 to 50%), while mesenchymal cells displayed little or no staining for ERBB4 (Figure 6). Solid tumor areas (CYT-1) contained neoplastic cells without any mammary ductal architecture, had necrotic regions, and displayed many scattered ERBB4 stained cells across the section. It is noteworthy that regions of ERBB4 expression contained highly proliferating cells, indicated by strong positive Ki-67 labeling (Figure 6). Furthermore, glandular carcinoma cells with higher percentage of ERBB4 also stained for STAT5a (50 to 100%) while tumor cells with squamous cell differentiation, and skeletal and smooth muscle cells stained negative for STAT5a. ErbB4 is known to bind and activate STAT5a and also increase its transcriptional activity [36]. To test whether ERBB4 induces epithelial–mesenchymal transition in mammary cells, we evaluated mesenchymal and epithelial markers. We found no evidence of epithelial–mesenchymal transition, as the protein expression patterns between luminal and myoepithelial cells were well demarcated by positive epithelial cell staining for E-cadherin while myoepithelial cells (mesenchymal) stained positive for vimentin, smooth muscle actin and CK5/6. Interestingly, mesenchymal cells within glandular carcinoma in CYT-1 mice showed very low p63 labeling, unlike other mesenchymal markers. CYT-1 glandular carcinoma cells expressing ERBB4, and STAT5a, and displaying Ki-67 staining, suggest a pro-proliferative role of ErbB4 in these tumor lesions. A recent study demonstrated that the ERBB3/ERBB4 ligand NRG1 is directly transcriptionally regulated by p63 expressed in myoepithelial cells, which induces luminal progenitor cell proliferation and milk production via paracrine ErbB4/STAT5a activation in luminal epithelium [37]. Since we found that p63 expression in CYT-1 expressing lesions is suppressed, this suggests a converse paracrine relation or feedback regulation between luminal epithelial ERBB4 and basal p63, wherein overexpression of CYT-1 isoform may be suppressing p63 expression. Masson trichrome stained collagen around ducts, keratin and muscle fibers present in connective tissue and nuclei. Synaptophysin, a marker for tumors of neuronal origin, was used as a negative control and, as expected, was negative in tumor tissue. F480, a macrophage marker, stained scattered macrophages in tumor lesions as expected (Additional file 3).

## Discussion

We developed transgenic mouse models overexpressing JM-a FL ERBB4 isoforms in mammary glands to determine the role of each isoform in mammary development and carcinogenesis. The FL CYT-1 isoform suppressed both mammary ductal elongation and branching in pubescent mice, with a concomitant reduction in the number of TEBs and the percentage of proliferating luminal cells in TEBs. FL CYT-2 isoform also mildly suppressed branching but did not affect ductal elongation in pubescent animals. The suppressive effect of CYT-1 expression on ductal branching was maintained in pregnant mice until mid-pregnancy, after which ERBB4 transgenic mice were developmentally similar and indistinguishable from controls. Finally, sustained expression of FL CYT-1 induced ERBB4-positive mammary tumor lesions in nearly all of the CYT-1 mice, indicating a tumorigenic function of this ERBB4 isoform in mammary epithelium.

Postnatal development of the mammary gland involves ductal and secretory phases that are regulated by concerted interplay of systemic hormones, locally secreted growth factors, and their receptors – including the EGFR family, which plays a major role [38],[39]. EGFR family members EGFR and ErbB2 are expressed abundantly and are active at all developmental stages, while ErbB3 and ErbB4 are mostly active during pregnancy and lactation. In the present study, the expression of ErbB4 isoforms was highest in pregnant and lactating mice. However, in pubescent mice – when ErbB4 expression is normally much lower – CYT-1 *ERBB4* transgene expression in mammary epithelium suppressed both mammary ductal elongation and branching, whereas the CYT-2 isoform had a suppressive effect on branching. The CYT-1 pubescent phenotype extended towards mid-pregnancy as CYT-1 isoform expression resulted in less dense mammary branching architecture until 12 days post coitus. We observed previously that mammary gland development of pubescent ErbB4 knockout mice outpaced the growth of sibling controls, which is consistent with a suppressive effect of ErbB4 [5]. However, this CYT-1 ERBB4 phenotype is in sharp contrast with the functions of EGFR, ErbB2, and ErbB3, which promote mammary ductal morphogenesis in pubescent mice [29]. Loss of function of either ErbB4, prolactin, prolactin receptor, JAK2, or STAT5 by various genetic methods results in similar phenotypes in mice characterized by impaired pregnant mammary lobuloalveolar growth and lactation defects [4],[40]-[42]. Impaired processing of ERBB4 ligand heparin-binding epidermal growth factor-like growth factor also impairs lactation [43]. Collectively, these data suggest that the branching and especially elongation phenotypes are mediated predominantly through EGFR, ErbB2, and ErbB3, when ErbB4 may play a minor suppressive function, whereas the pro-differentiation phenotypes manifested late in pregnancy and during lactation are mediated through ErbB4.

Suppressed ductal morphogenesis by CYT-1 ERBB4 may result from the smaller number of proliferating cells observed within the CYT-1 TEBs. This effect on cell proliferation could be mediated through both cellular and tissue-level effects of ERBB4 signaling. *In vitro*, the transforming growth factor-beta pathway has been shown to be upregulated by ErbB4 activation in T47D and MCF10A mammary carcinoma cells [25],[44]; *in vivo*, epithelial transforming growth factor-beta inhibits forward movement of TEBs [45]. Although activated ERBB4 has been shown to induce apoptosis [46], we found no evidence of apoptosis, as indicated by the absence of cleaved caspase-3 immunoreactivity (data not shown). The unique activities of ErbB4 are mediated partly through activities of cleaved nuclear isoforms that affect transcription through binding to Stat5, YAP, and other transcriptional regulators [25],[47]. Also, luminal cells were loosely packed in 8-week MMTV-CYT-1 TEBs. This may be relevant to the phenotype, as disruption of cell–cell contacts within TEBs inhibits cell proliferation and ductal growth [48].

GFP-tagged ICD CYT-1 and CYT-2 have been expressed in mouse mammary glands using a doxycycline-inducible transgenic model [23], in which CYT-2 increased while CYT-1 decreased pubertal growth of mammary ducts. CYT-1 mice had fewer TEBs with lower proliferative index, while CYT-2 mice had more TEBs with higher fraction of proliferating cells. While biologically interesting, this mouse model does not fully recapitulate the physiological activity of FL ERBB4. Unlike cleaved ICDs that are constitutively active and equally capable of entering the nucleus, FL ERBB4 must undergo a two-step cleavage event at the membrane to generate cytoplasmic and nuclear pools of ERBB4. Our FL mouse model expressing FL ERBB4 brings in this additional level of regulation by endogenous agonists and metalloproteinases at physiologically relevant levels. Comparing our FL model with the previously described ICD model allows us to speculate on the relative contribution of ERBB4 at the membrane. Both FL ERBB4 CYT-1 mice and CYT-1 ICD mice showed similar phenotypes of suppressed ductal elongation with decreased numbers of TEBs and proliferating cells, but ICD CYT-1 did not suppress ductal branching in pubertal or pregnant mice. In contrast with FL CYT-2, which had no effect on TEBs and a slight suppressive effect on branching, ICD CYT-2 mice showed an increase in TEBs and cellular proliferation and hyperplasia. These observations suggest that CYT-1 ICD mirrors FL CYT-1 function more so than CYT-2 ICD recapitulates FL CYT-2 during mammary development. The major difference between FL and ICD ERBB4 developmental phenotypes is the inhibition of mammary ductal branching, only seen with FL ERBB4 isoforms, which may be due to membrane-associated functions of FL ERBB4.

Overexpression of ErbB1, ErbB2, and ErbB3 in transgenic mouse models contributes to mammary tumor formation. Mammary gland-specific human EGFR transgene expression under MMTV long terminal repeat induces neoplasia in mice [49]. Similarly, elevated expression of activated forms of Neu/ErbB-2 and ErbB3 are involved in the induction of mammary tumors in MMTV-Neu transgenic mice [50]. In contrast, transgenic expression of truncated ERBB4 isoforms did not induce neoplasia [23]. But sustained expression of the FL CYT-1 ERBB4 isoform resulted in the formation of neoplastic lesions/tumor in the present study, while CYT-2 expression only caused a low incidence of hyperplasia, hinting at a possible milder oncogenic predisposition of CYT-2. CYT-1 ERBB4-induced tumors may develop either directly by sustained and enhanced downstream ERBB4 signaling in a FVB genetic background, or from ERBB4 signaling acting in concert with secondary genetic or epigenetic alterations acquired over a 1-year period. Nonetheless, the similar tumor characteristics in all CYT-1 mice suggest the former rather than the latter. Multiparous transgenic female mice expressing NRG1, a ligand for ERBB3 and ERBB4, under control of the MMTV promoter also develop adenocarcinomas in the mammary glands at a median age of 12 months [51], suggesting ErbB4 to be contributing to neuregulin-induced carcinogenesis. In the composite ErbB signaling network, whether overexpressed CYT-1 induces expression of agonists such as neuregulins and/or interacts with ERBB3 and ERBB2 more efficiently, enabling it to be tumorigenic, awaits further investigation. Notably, it was recently shown that expression of CYT-1 ERBB4 is associated with poor survival from ovarian cancer [24]. Analysis of mammary gland mRNA expression of the CYT-1 and CYT-2 isoforms also indicates a higher ratio of CYT-1:CYT-2 expression in breast cancers (>50% CYT-1) versus normal mammary glands (<40% CYT-1) [1]. Expression of FL ERBB4 probably creates more complex phenotypes arising from the dynamic state of the receptor (membranous, nuclear, or cytoplasmic), extracellular interactions, and a milieu of endogenous agonists that are differentially expressed (NRG1, heparin-binding epidermal growth factor-like growth factor, betacellulin, and so forth) that probably contribute to the phenotypes we observed.

## Conclusions

These ERBB4 mouse models are the first to describe overexpression of ERBB4 in noncardiac tissue. They are especially significant as ERBB4 overexpression is more frequent than ERBB4 mutations in cancer, but the biological impact is uncertain. The mammary phenotypes resulting from FL ERBB4 expression most closely model complex ERBB4 functions resulting from composite signaling by intact and truncated (s80) ERBB4. Results of the present study demonstrate different functional roles of ERBB4 isoforms in mammary development, and mammary tumorigenesis. ERBB4 CYT-1 initially suppresses mammary ductal morphogenesis, but eventually this defect is corrected later in development. We describe a causal relation between CYT-1 ERBB4 expression and tumorigenesis in transgenic mice. Together, these results significantly expand the understanding of ErbB4 function in early developmental phases when its expression is low, and reveal novel oncogenic properties of ERBB4 CYT-1 isoform. These findings also suggest that it might be advantageous to inhibit ERBB4 specifically or use pan-ERBB inhibitors for treatment of certain subsets of breast cancer.

## Additional files

## Electronic supplementary material

Additional file 1: Figures S1 to S8 showing left-side #4 mammary glands isolated from female transgenic and sibling FVB control mice for whole mount staining with Carmine Alum: 5 weeks virgin (Figure S1), 8 weeks virgin (Figure S2), 14 weeks virgin (Figure S3), 12 days post-coitus CYT-1 (Figure S4), 12 days post-coitus CYT-2 (Figure S5), 19 days post-coitus (Figure S6), 1 day post-partum (Figure S7), and 16 days post-weaning (Figure S8). Entire glands were photographed under a dissection microscope with a SPOT 11.2 Color Mosaic camera (Diagnostic Instruments Inc.) at 10× magnification using SPOT advanced software 4.0.9, and analyzed. (ZIP 28 MB)

Additional file 2: Figure S9 showing whole mounts of mammary glands from nonpregnant age-matched (52-week) multiparous female control, CYT-1 and CYT-2 mice removed from glass slides, embedded in paraffin sections and stained by H&E by routine methods for verification of pathologic findings compared with control (A) mice. CYT-1 mice mammary glands had the following pathologic changes: mild glandular hyperplasia (B), moderate glandular hyperplasia (C), or neoplasia (D to N). Neoplasia varied from single small adenomas within hyperplasic regions, diffuse areas with ductular hyperplasia (E) to discrete adenocarcinomas (F to N) of which many had foci of squamous differentiation (G, K, M, N) and frequent inflammatory cells (H, K, L, N). Two out of 12 CYT-2 mice (O to Q) also had lesions but fewer than CYT-1 mice, and pathologic changes were similar: glandular hyperplasia (O), adenocarcinoma (P), and squamous differentiation (Q). Scale bars = 50 μm. (PPTX 2 MB)

Additional file 3: Figure S10 showing mammary tissue isolated from age-matched (52-week) female multiparous control FVB (normal), abnormal regions observed in CYT-2 (hyperplasia), and CYT-1 (adenocarcinoma, squamous differentiation, solid tumor) ERBB4 transgenic mice, embedded in paraffin sections and processed for immunohistochemistry to stain for synaptophysin (Synap), a marker for tissues of neuronal origin, and F/480 (F480), which stains macrophages. Scale bars = 50 μm. (PPTX 643 KB)

Below are the links to the authors’ original submitted files for images.

Authors’ original file for figure 1

Authors’ original file for figure 2

Authors’ original file for figure 3

Authors’ original file for figure 4

Authors’ original file for figure 5

Authors’ original file for figure 6

## Abbreviations

EGFR: epidermal growth factor receptor
FL: full length
H&E: hematoxylin and eosin
ICD: intracellular domain
IHC: immunohistochemistry
MMTV: mouse mammary tumor virus
NRG1: Neuregulin 1
PCNA: proliferating cell nuclear antigen
STAT5: signal transducer and activator of transcription 5
TEB: terminal end bud
