# Supplementary material for: Overexpression of ERBB4 JM-a CYT-1 and CYT-2 isoforms in transgenic mice reveals isoform-specific roles in mammary gland development and carcinogenesis
Source: Breast Cancer Res. 2014 Dec 17;16:501. doi: 10.1186/s13058-014-0501-z (PMC4303208; doi:10.1186/s13058-014-0501-z)
Supplement: Supplementary file 1 — Additional file 1: Figures S1 to S8 showing left-side #4 mammary glands isolated from female transgenic and sibling FVB control mice for whole mount staining with Carmine Alum: 5 weeks virgin (Figure S1), 8 weeks virgin (Figure S2), 14 weeks virgin (Figure S3), 12 days post-coitus CYT-1 (Figure S4), 12 days post-coitus CYT-2 (Figure S5), 19 days post-coitus (Figure S6), 1 day post-partum (Figure S7), and 16 days post-weaning (Figure S8). Entire glands were photographed under a dissection microscope with a SPOT 11.2 Color Mosaic camera (Diagnostic Instruments Inc.) at 10× magnification using SPOT advanced software 4.0.9, and analyzed. (ZIP 28 MB) [file 13058_2014_501_MOESM1_ESM.zip › 2107022569132648_add7.pptx]

## Slide 1
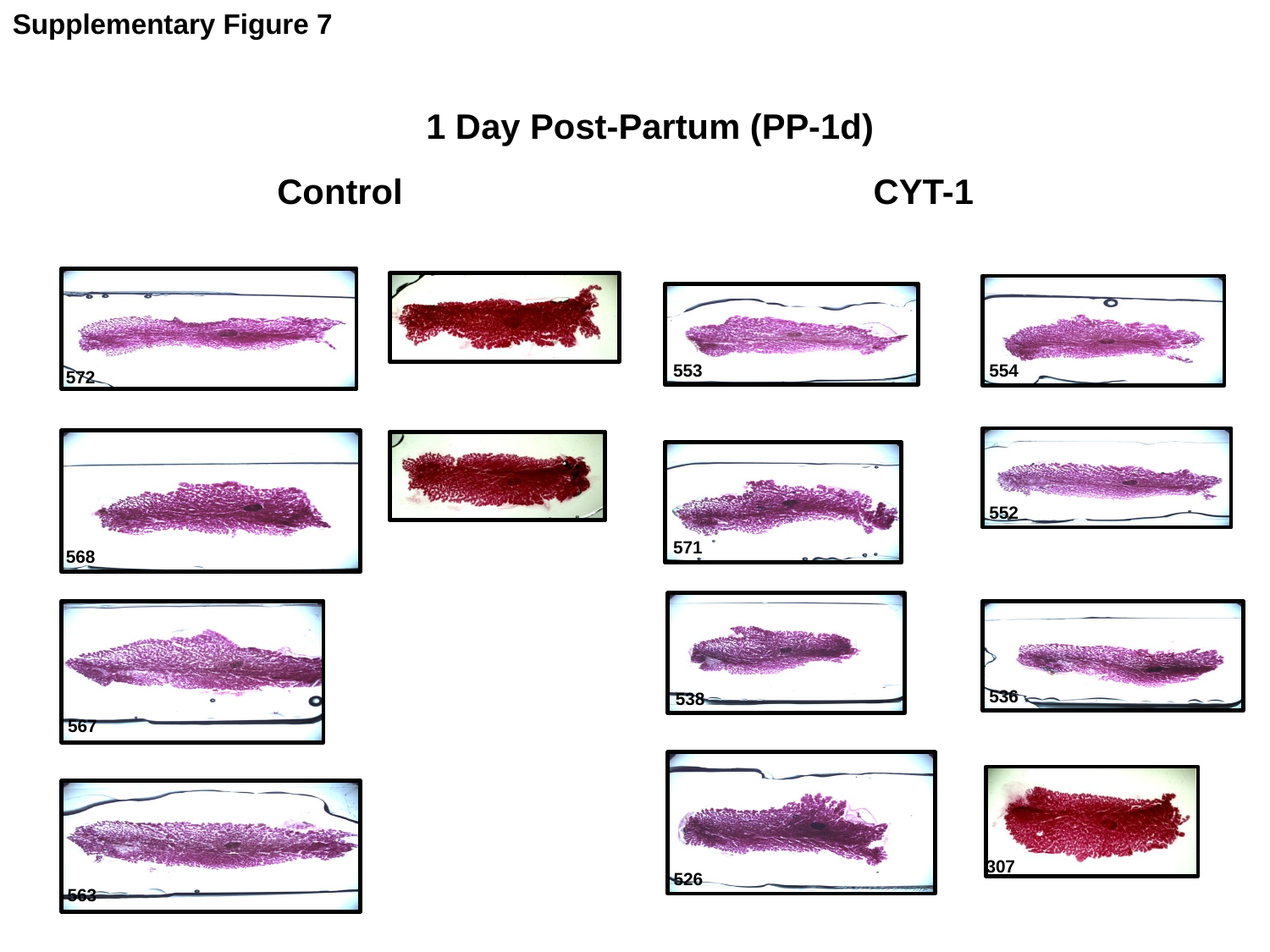

Supplementary Figure 7
1 Day Post-Partum (PP-1d)
Control
CYT-1
553
554
572
552
571
568
536
538
567
307
526
563

## Slide 2
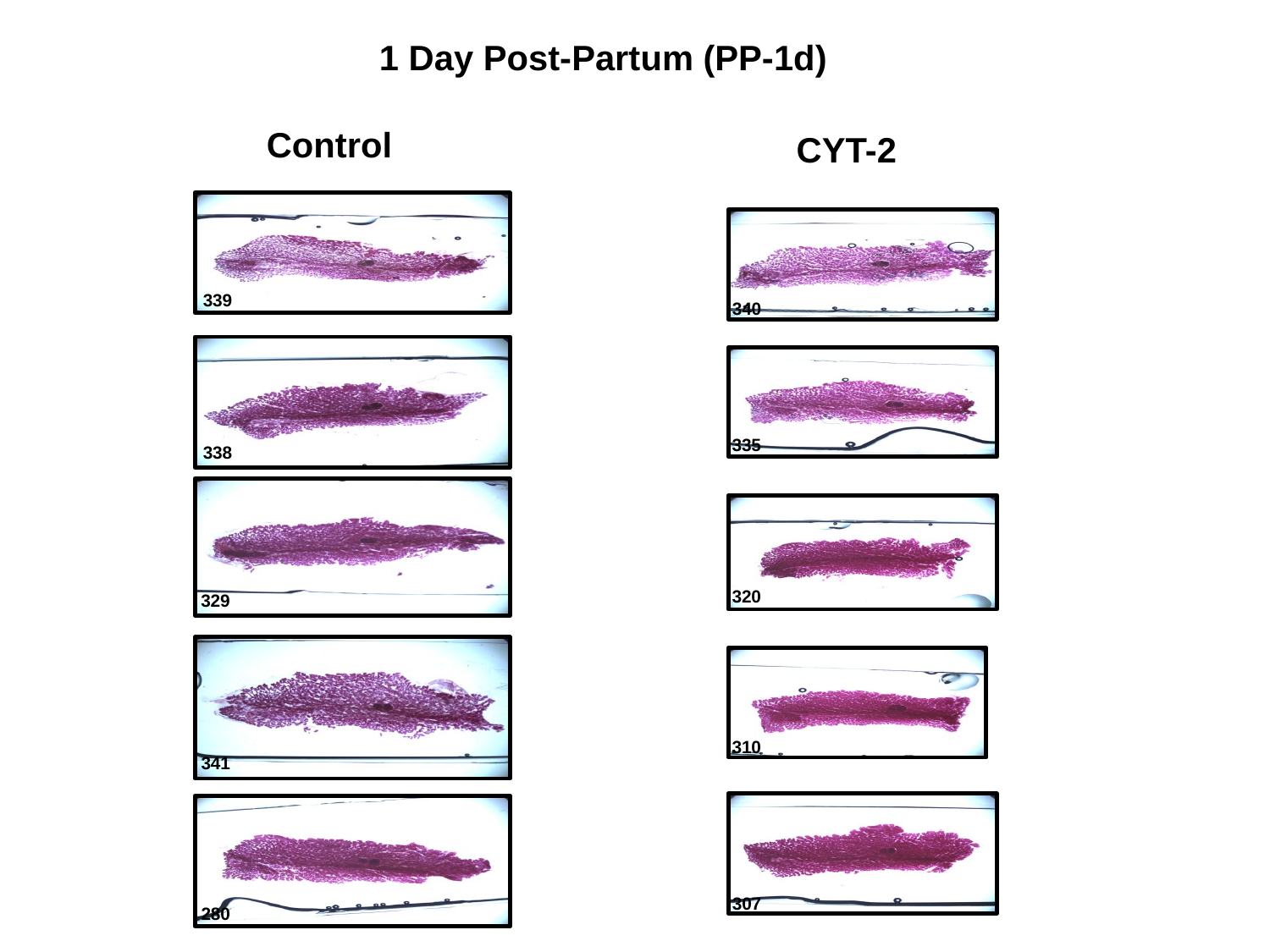

1 Day Post-Partum (PP-1d)
Control
CYT-2
339
340
335
338
320
329
310
341
307
280
