# Supplementary figures and images for: Overexpression of ERBB4 JM-a CYT-1 and CYT-2 isoforms in transgenic mice reveals isoform-specific roles in mammary gland development and carcinogenesis
Source: Breast Cancer Res. 2014 Dec 17;16:501. doi: 10.1186/s13058-014-0501-z (PMC4303208; doi:10.1186/s13058-014-0501-z)

## Slide 1
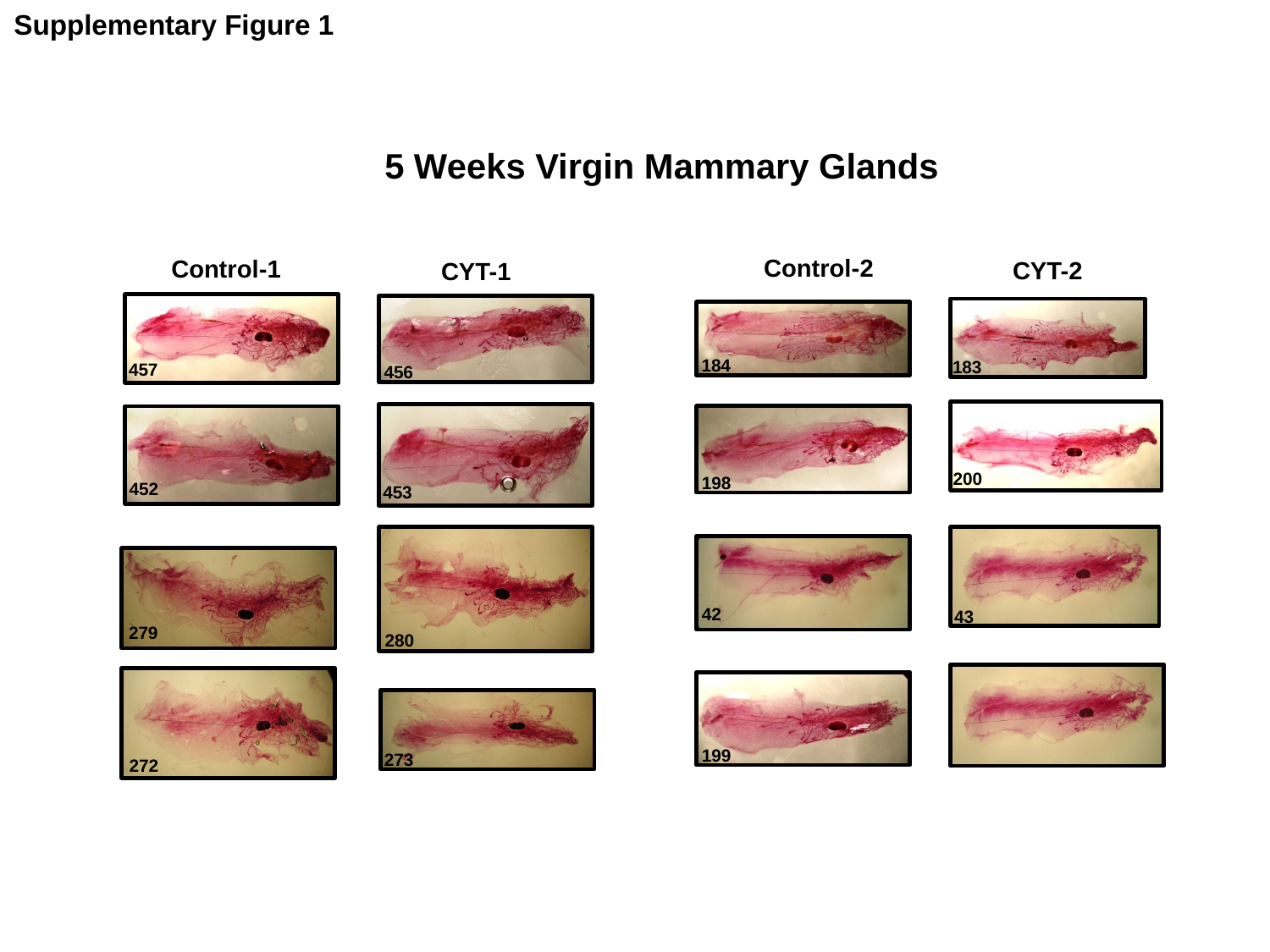

Supplementary Figure 1
5 Weeks Virgin Mammary Glands
Control-2
Control-1
CYT-2
CYT-1
184
183
457
456
200
198
452
453
42
43
279
280
199
273
272

Supplement: Supplementary file 1 — Additional file 1: Figures S1 to S8 showing left-side #4 mammary glands isolated from female transgenic and sibling FVB control mice for whole mount staining with Carmine Alum: 5 weeks virgin (Figure S1), 8 weeks virgin (Figure S2), 14 weeks virgin (Figure S3), 12 days post-coitus CYT-1 (Figure S4), 12 days post-coitus CYT-2 (Figure S5), 19 days post-coitus (Figure S6), 1 day post-partum (Figure S7), and 16 days post-weaning (Figure S8). Entire glands were photographed under a dissection microscope with a SPOT 11.2 Color Mosaic camera (Diagnostic Instruments Inc.) at 10× magnification using SPOT advanced software 4.0.9, and analyzed. (ZIP 28 MB) [file 13058_2014_501_MOESM1_ESM.zip › 2107022569132648_add1.pptx]

## Slide 1
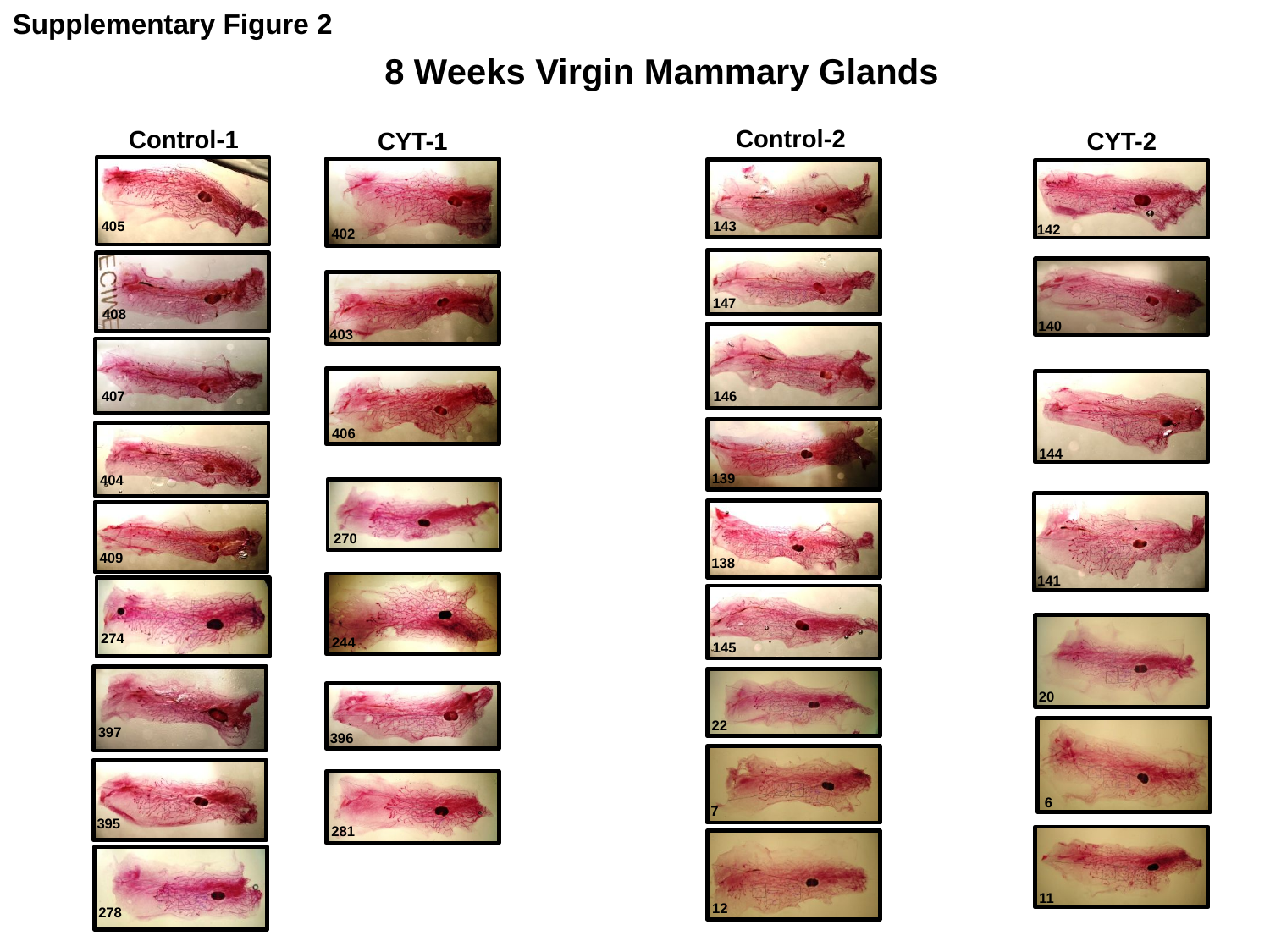

Supplementary Figure 2
8 Weeks Virgin Mammary Glands
Control-2
Control-1
CYT-2
CYT-1
143
405
142
402
147
408
140
403
146
407
406
144
139
404
270
409
138
141
274
244
145
20
22
397
396
6
7
395
281
11
12
278

Supplement: Supplementary file 1 — Additional file 1: Figures S1 to S8 showing left-side #4 mammary glands isolated from female transgenic and sibling FVB control mice for whole mount staining with Carmine Alum: 5 weeks virgin (Figure S1), 8 weeks virgin (Figure S2), 14 weeks virgin (Figure S3), 12 days post-coitus CYT-1 (Figure S4), 12 days post-coitus CYT-2 (Figure S5), 19 days post-coitus (Figure S6), 1 day post-partum (Figure S7), and 16 days post-weaning (Figure S8). Entire glands were photographed under a dissection microscope with a SPOT 11.2 Color Mosaic camera (Diagnostic Instruments Inc.) at 10× magnification using SPOT advanced software 4.0.9, and analyzed. (ZIP 28 MB) [file 13058_2014_501_MOESM1_ESM.zip › 2107022569132648_add2.pptx]

## Slide 1
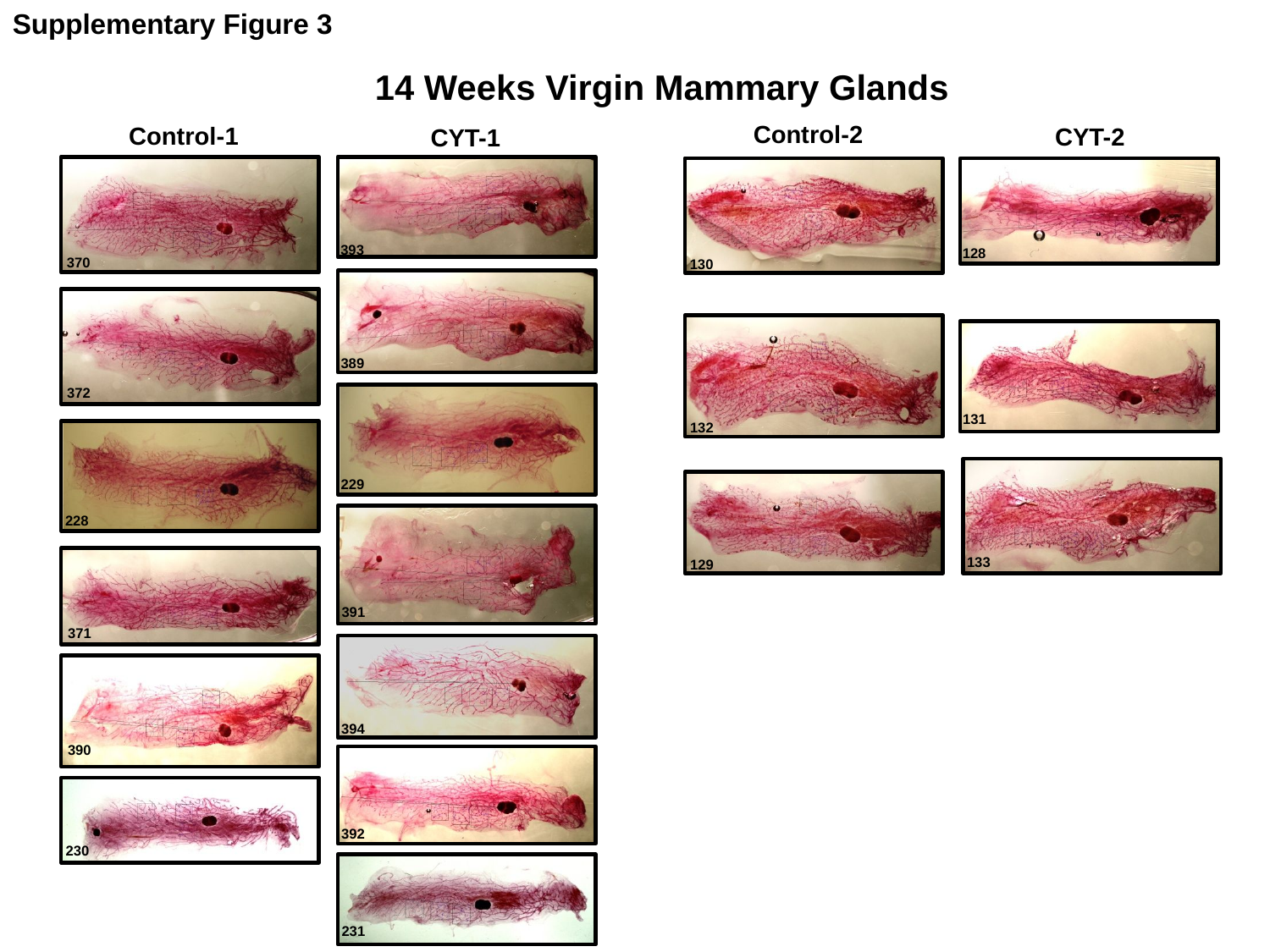

Supplementary Figure 3
14 Weeks Virgin Mammary Glands
Control-2
Control-1
CYT-2
CYT-1
393
128
370
130
389
372
131
132
229
228
133
129
391
371
394
390
392
230
231

Supplement: Supplementary file 1 — Additional file 1: Figures S1 to S8 showing left-side #4 mammary glands isolated from female transgenic and sibling FVB control mice for whole mount staining with Carmine Alum: 5 weeks virgin (Figure S1), 8 weeks virgin (Figure S2), 14 weeks virgin (Figure S3), 12 days post-coitus CYT-1 (Figure S4), 12 days post-coitus CYT-2 (Figure S5), 19 days post-coitus (Figure S6), 1 day post-partum (Figure S7), and 16 days post-weaning (Figure S8). Entire glands were photographed under a dissection microscope with a SPOT 11.2 Color Mosaic camera (Diagnostic Instruments Inc.) at 10× magnification using SPOT advanced software 4.0.9, and analyzed. (ZIP 28 MB) [file 13058_2014_501_MOESM1_ESM.zip › 2107022569132648_add3.pptx]

## Slide 1
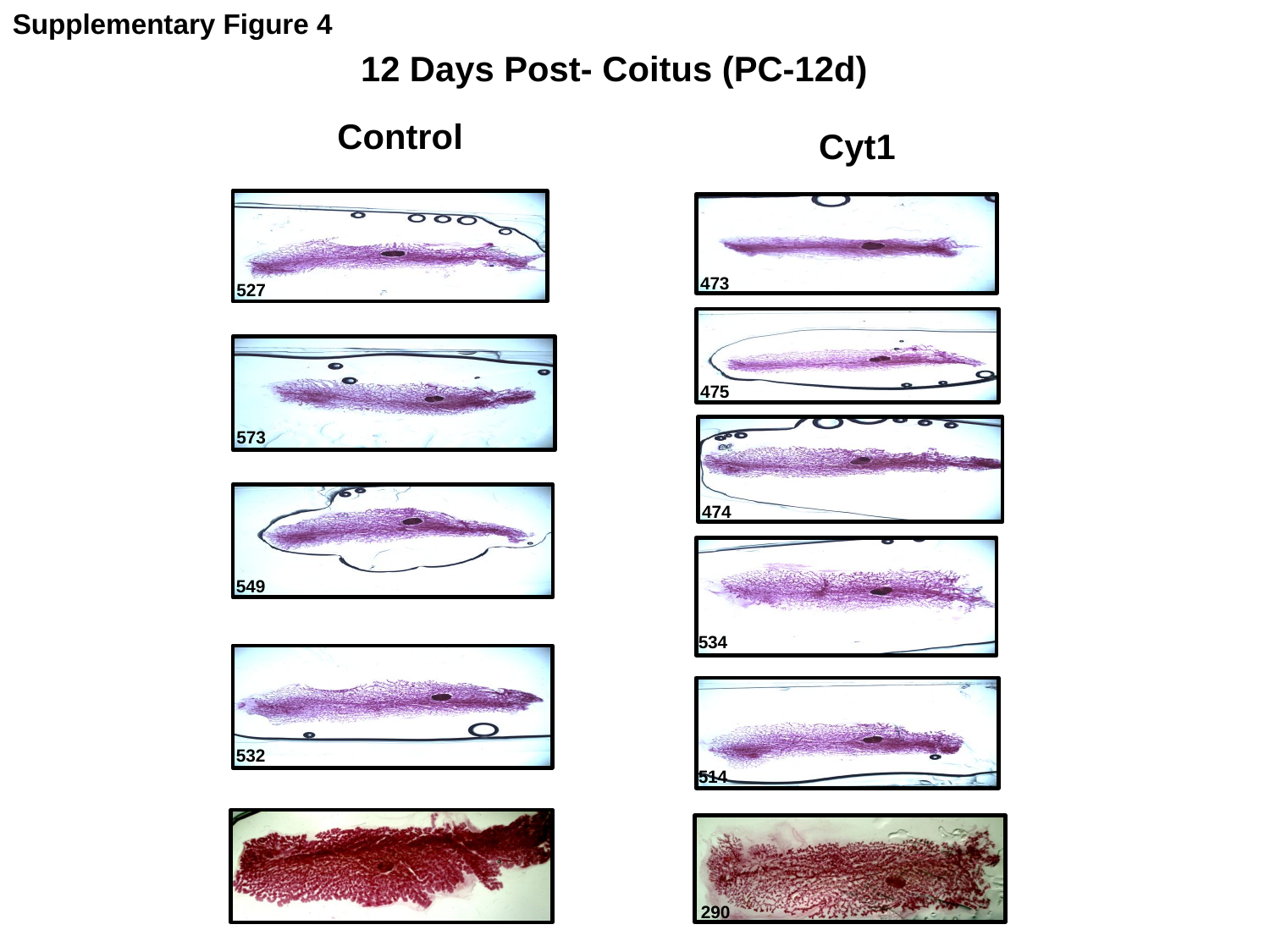

Supplementary Figure 4
12 Days Post- Coitus (PC-12d)
Control
Cyt1
473
527
475
573
474
549
534
532
514
290

Supplement: Supplementary file 1 — Additional file 1: Figures S1 to S8 showing left-side #4 mammary glands isolated from female transgenic and sibling FVB control mice for whole mount staining with Carmine Alum: 5 weeks virgin (Figure S1), 8 weeks virgin (Figure S2), 14 weeks virgin (Figure S3), 12 days post-coitus CYT-1 (Figure S4), 12 days post-coitus CYT-2 (Figure S5), 19 days post-coitus (Figure S6), 1 day post-partum (Figure S7), and 16 days post-weaning (Figure S8). Entire glands were photographed under a dissection microscope with a SPOT 11.2 Color Mosaic camera (Diagnostic Instruments Inc.) at 10× magnification using SPOT advanced software 4.0.9, and analyzed. (ZIP 28 MB) [file 13058_2014_501_MOESM1_ESM.zip › 2107022569132648_add4.pptx]

## Slide 1
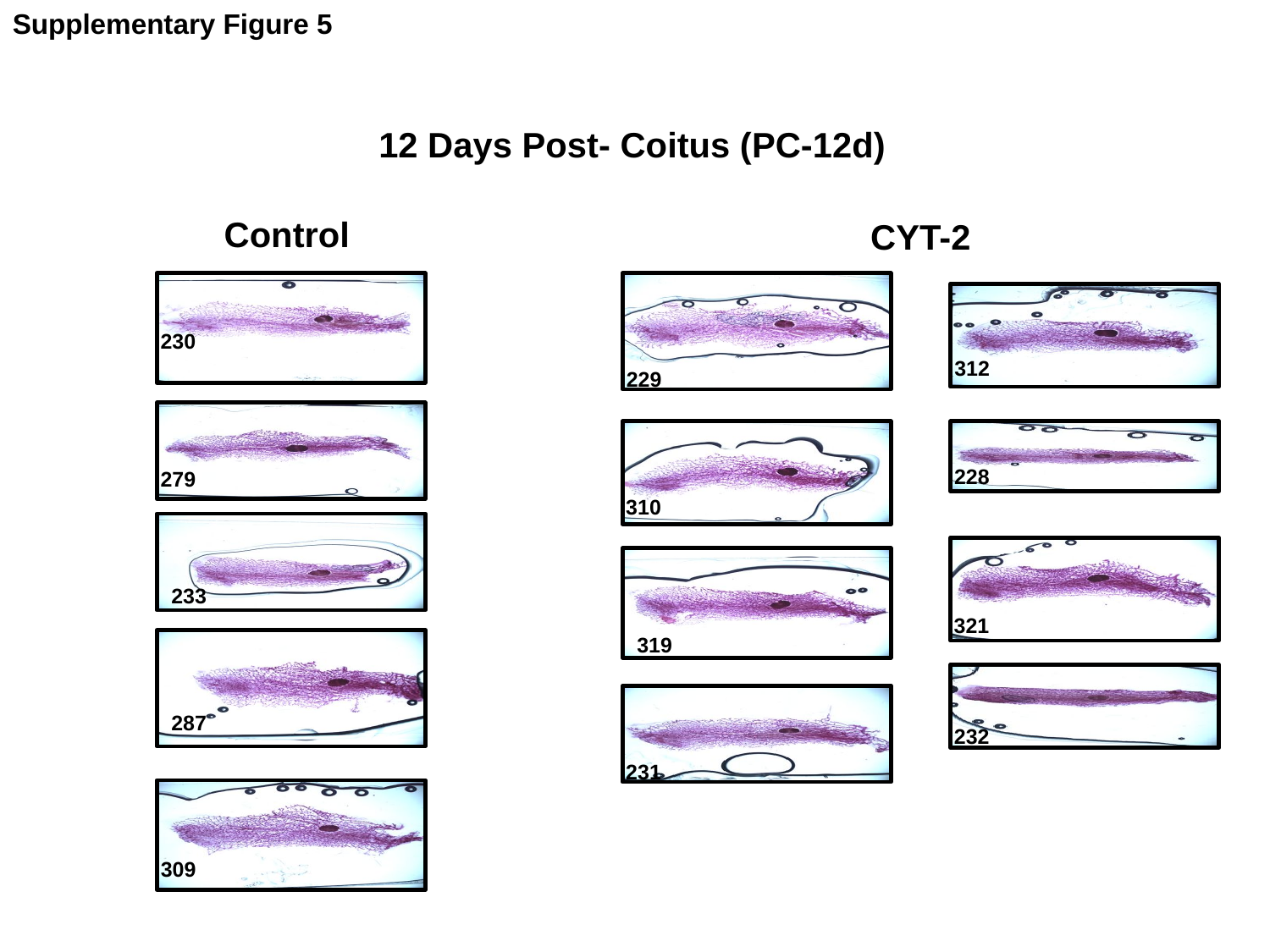

Supplementary Figure 5
12 Days Post- Coitus (PC-12d)
Control
CYT-2
230
312
229
228
279
310
233
321
319
287
232
231
309

Supplement: Supplementary file 1 — Additional file 1: Figures S1 to S8 showing left-side #4 mammary glands isolated from female transgenic and sibling FVB control mice for whole mount staining with Carmine Alum: 5 weeks virgin (Figure S1), 8 weeks virgin (Figure S2), 14 weeks virgin (Figure S3), 12 days post-coitus CYT-1 (Figure S4), 12 days post-coitus CYT-2 (Figure S5), 19 days post-coitus (Figure S6), 1 day post-partum (Figure S7), and 16 days post-weaning (Figure S8). Entire glands were photographed under a dissection microscope with a SPOT 11.2 Color Mosaic camera (Diagnostic Instruments Inc.) at 10× magnification using SPOT advanced software 4.0.9, and analyzed. (ZIP 28 MB) [file 13058_2014_501_MOESM1_ESM.zip › 2107022569132648_add5.pptx]

## Slide 1
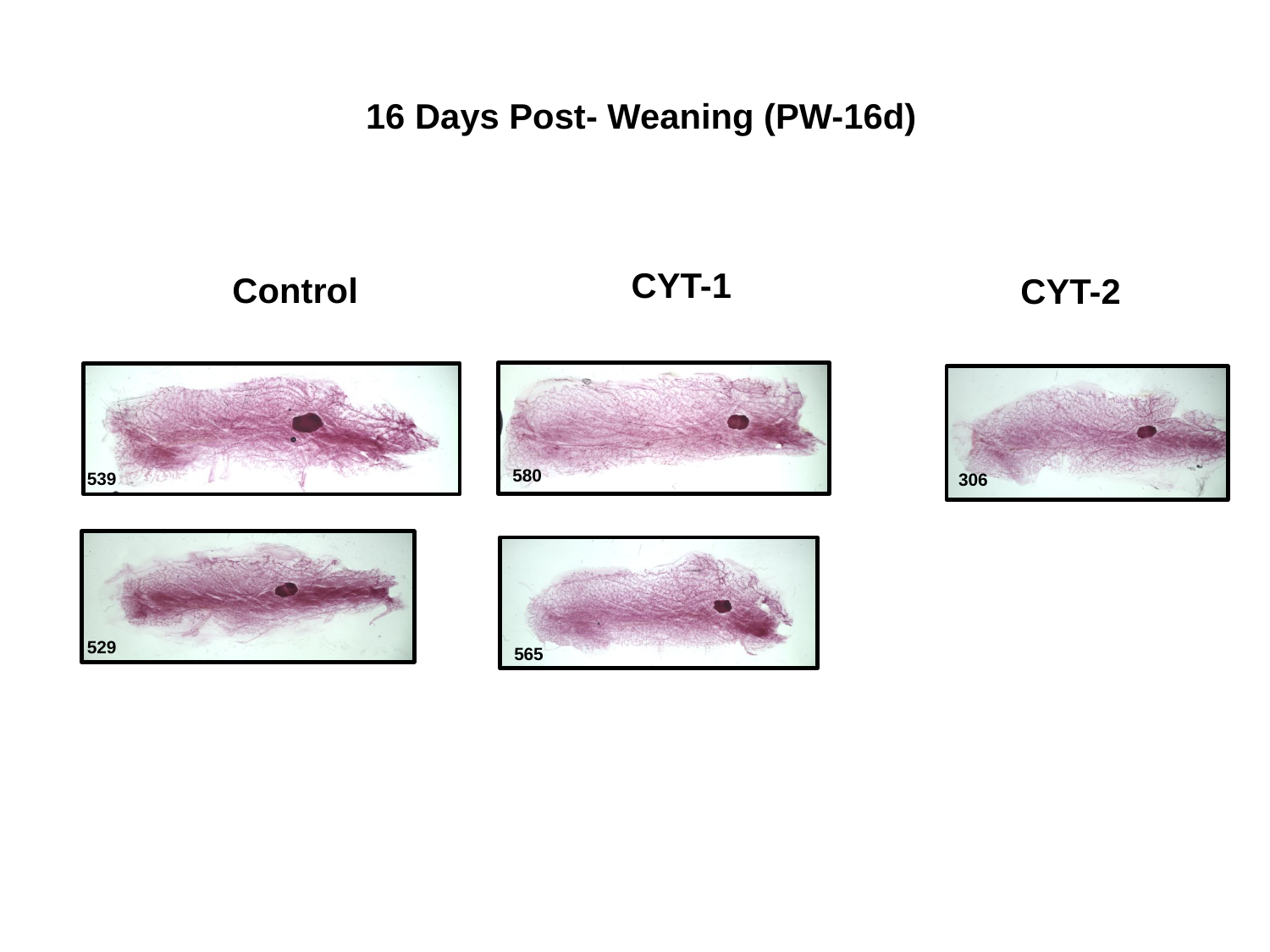

16 Days Post- Weaning (PW-16d)
CYT-1
Control
CYT-2
580
539
306
529
565

Supplement: Supplementary file 1 — Additional file 1: Figures S1 to S8 showing left-side #4 mammary glands isolated from female transgenic and sibling FVB control mice for whole mount staining with Carmine Alum: 5 weeks virgin (Figure S1), 8 weeks virgin (Figure S2), 14 weeks virgin (Figure S3), 12 days post-coitus CYT-1 (Figure S4), 12 days post-coitus CYT-2 (Figure S5), 19 days post-coitus (Figure S6), 1 day post-partum (Figure S7), and 16 days post-weaning (Figure S8). Entire glands were photographed under a dissection microscope with a SPOT 11.2 Color Mosaic camera (Diagnostic Instruments Inc.) at 10× magnification using SPOT advanced software 4.0.9, and analyzed. (ZIP 28 MB) [file 13058_2014_501_MOESM1_ESM.zip › 2107022569132648_add8.pptx]

## Slide 1
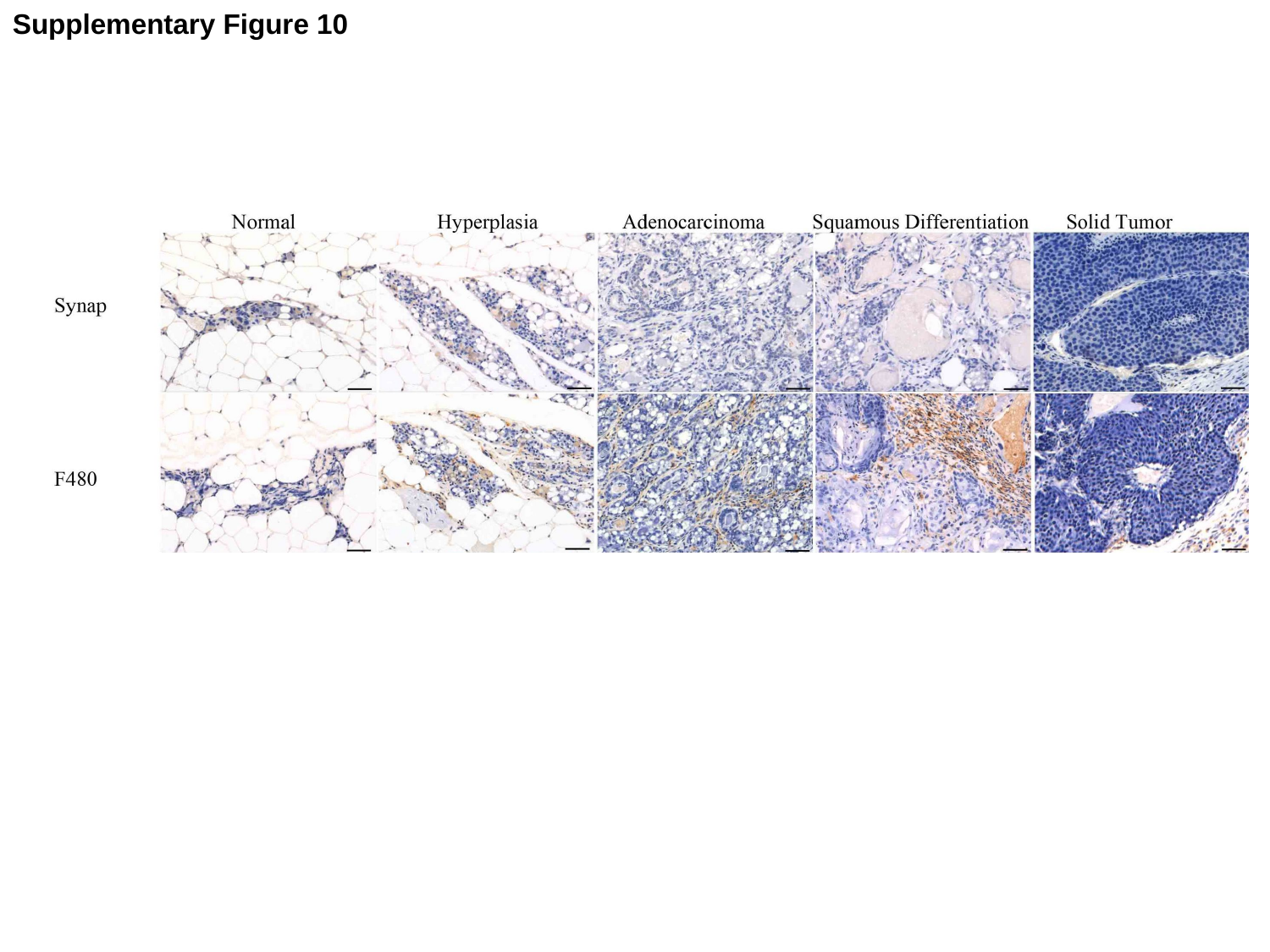

Supplementary Figure 10

Supplement: Supplementary file 3 — Additional file 3: Figure S10 showing mammary tissue isolated from age-matched (52-week) female multiparous control FVB (normal), abnormal regions observed in CYT-2 (hyperplasia), and CYT-1 (adenocarcinoma, squamous differentiation, solid tumor) ERBB4 transgenic mice, embedded in paraffin sections and processed for immunohistochemistry to stain for synaptophysin (Synap), a marker for tissues of neuronal origin, and F/480 (F480), which stains macrophages. Scale bars = 50 μm. (PPTX 643 KB) [file 13058_2014_501_MOESM3_ESM.pptx]

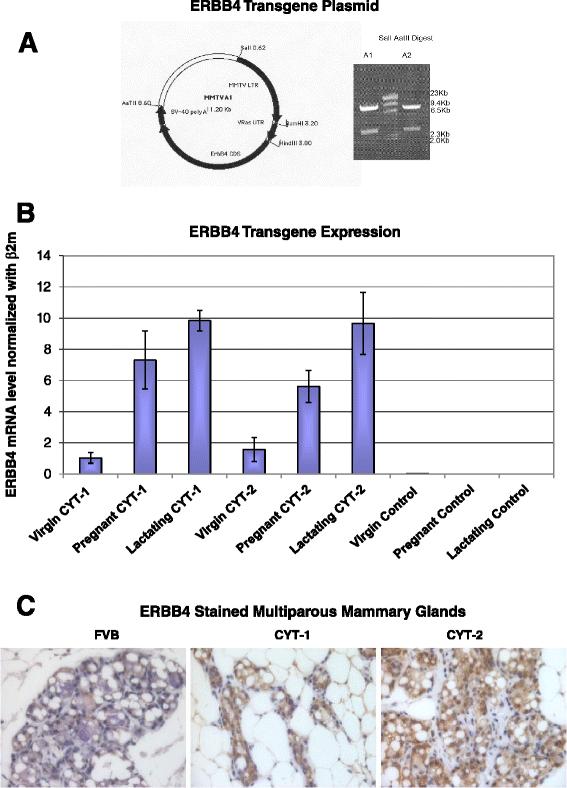

Supplement: Supplementary file 4 — Authors’ original file for figure 1 [file 13058_2014_501_MOESM4_ESM.gif]

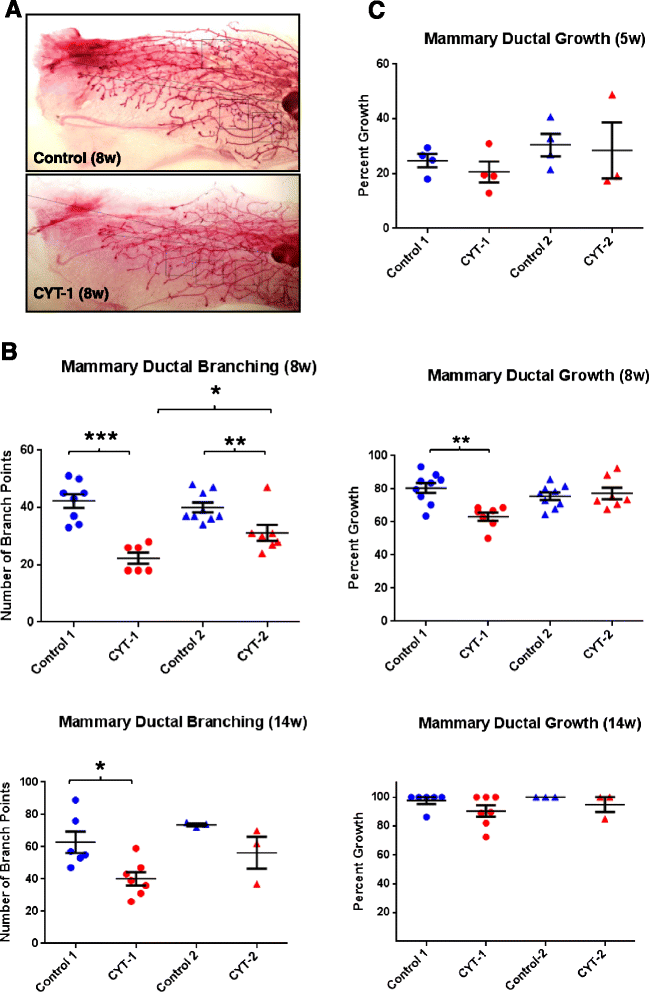

Supplement: Supplementary file 5 — Authors’ original file for figure 2 [file 13058_2014_501_MOESM5_ESM.gif]

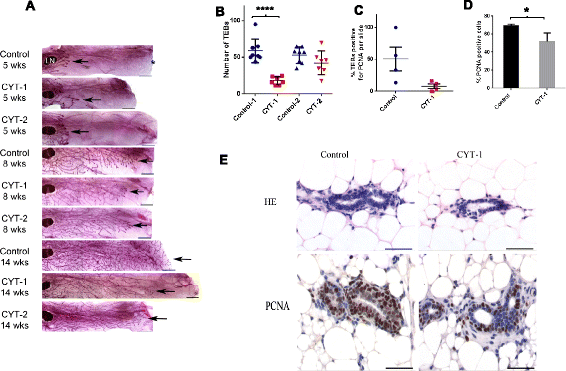

Supplement: Supplementary file 6 — Authors’ original file for figure 3 [file 13058_2014_501_MOESM6_ESM.gif]

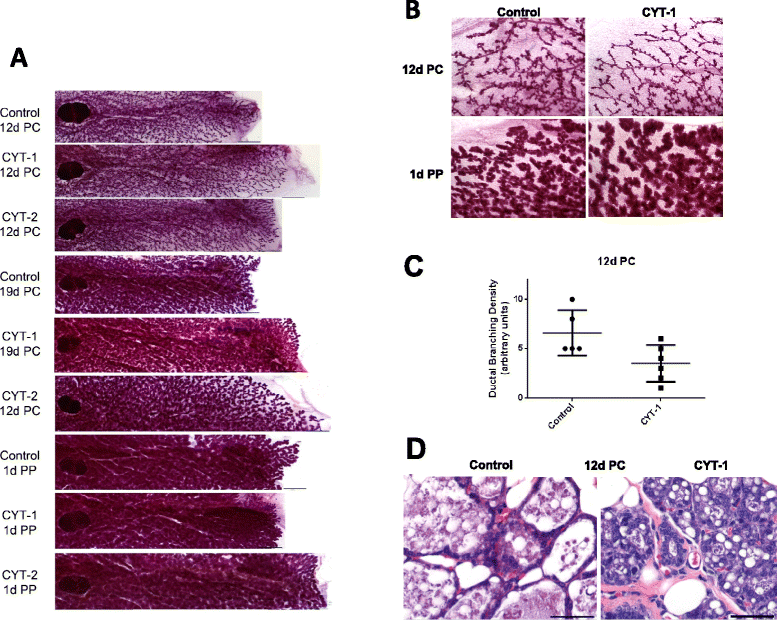

Supplement: Supplementary file 7 — Authors’ original file for figure 4 [file 13058_2014_501_MOESM7_ESM.gif]

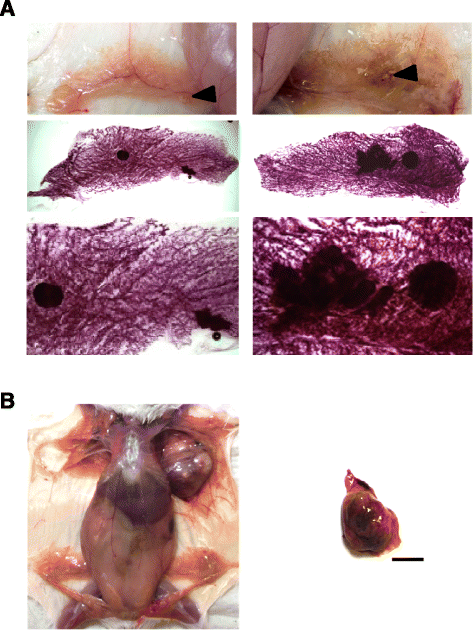

Supplement: Supplementary file 8 — Authors’ original file for figure 5 [file 13058_2014_501_MOESM8_ESM.gif]

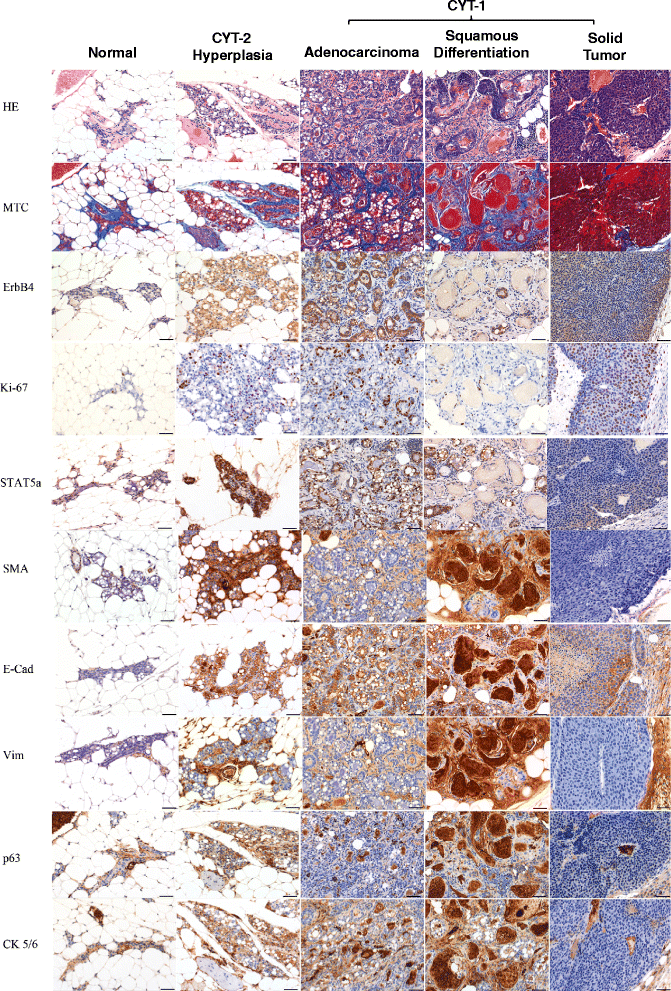

Supplement: Supplementary file 9 — Authors’ original file for figure 6 [file 13058_2014_501_MOESM9_ESM.gif]
